# The relationship between Big Five Personality Traits, eating habits, physical activity, and obesity in Indonesia based on analysis of the 5th wave Indonesia Family Life Survey (2014)

**DOI:** 10.3389/fpsyg.2022.881436

**Published:** 2022-08-04

**Authors:** Greena Pristyna, Trias Mahmudiono, Mahmud A. Rifqi, Diah Indriani

**Affiliations:** ^1^Department of Nutrition, Faculty of Public Health, Airlangga University, Surabaya, Indonesia; ^2^Department of Biostatistics and Medical Demography, Faculty of Public Health, Airlangga University, Surabaya, Indonesia

**Keywords:** Big Five Personality Traits, physical activity, obesity, Body Mass Index, Indonesia, eating habits

## Abstract

This study investigated the association between Big Five Personality Traits (Openness to new experience, Conscientiousness, Extraversion, Agreeableness, Neuroticism) and nutrition-related variables (eating habits, physical activity, and obesity using Body Mass Index). We used secondary data from the Indonesia Family Life Survey (IFLS) wave 5 involving a total of 14,473 men and 16,467 women aged 15−101 years (mean = 37.34; *SD* = 14.916) in Indonesia that was selected by stratified random sampling conducted in the period 2014 to 2015. Data were collected through interviews with the Big Five Index 15 and a questionnaire similar to the Global Physical Activity Questionnaire which was translated into the Indonesian language, and based on measurements of height and weight. Analyses used binary logistic regression test controlled by socio-demographic factors (age, gender, education, occupation, and marital status) to determine the association between Big Five Personality Traits and eating habits (recommended and non-recommended foods), physical activity, and obesity. Results showed that openness and extraversion were positively associated with recommended and non-recommended foods, conscientiousness was positively associated with only recommended foods, agreeableness was positively associated with recommended foods, and negatively associated with only one non-recommended food. Whereas, neuroticism was positively associated with non-recommended foods and negatively associated with recommended foods. Openness (*p* = 0.010; OR = 1.015; 95% CI = 1.004−1.027) and conscientiousness (*p* < 0.001; OR = 1.045; 95% CI = 1.030−1.059) were associated with physical activity. Conscientiousness (*p* = 0.002; OR = 1.025; 95% CI = 1.009−1.041), extraversion (*p* < 0.001; OR = 1.079; 95% CI = 1.065−1.092), and neuroticism (*p* < 0.001; OR = 0.966; 95% CI = 0.953−0.978) were associated with obesity. Although some results were different from previous studies, these associations can be used as strategies of behavioral change due to the adaptation of personality characteristics, which can be modified even though the personality tends to be persistent. Further studies are needed to understand other mechanisms that might underlie this association.

## Introduction

Individuals need to maintain their health by showing healthy behavior in order to avoid various health problems. Poor eating habits can have a negative effect on an individual’s nutritional status (Indonesian Ministry of Health [IMH], 2017); furthermore, a study published by The Lancet journal found that the food consumed daily was the leading cause of death compared to smoking and now has become one of the five causes of death worldwide (Indonesian Ministry of Health [IMH], 2019). Moreover, lack of physical activity is also a major risk factor for chronic diseases and premature death due to non-communicable diseases (World Health Organization [WHO], 2018), although regular physical activity can not only decrease the risk of various diseases but also can improve mental health and quality of life (World Health Organization [WHO], 2018).

Personality is a dynamic and complex physical and psychological system within the individual that underlies every behavior (Ahdiyana, 2011; Riadi, 2012). Personality can be associated with several health conditions that can be explained by personality-related behavioral factors (Tiainen et al., 2013). As one of the personality theories that is often used, the Big Five Personality Traits or Five-Factor Model (FFM) developed by Costa and McCrae (1985) divided human personality into five major dimensions: Agreeableness (A), Extraversion (E), Neuroticism (N), Conscientiousness (C), and Openness to experience (O) (Feist et al., 2013), which were associated with several health-related behaviors.

Previous studies found an association between personality, particularly Big Five Personality Traits with nutrition-related behaviors (such as eating habits and physical activity) and nutritional status, but most of these studies were conducted in Western countries, such as the study by Sutin et al. (2011) conducted in America, Terracciano et al. (2009) in Italy, Intiful et al. (2019) in Ghana, the study by Tiainen et al. (2013) were conducted on people born in Helsinki, Finland, the meta-analysis by Sutin et al. (2016) consisting of the United States, England, and Japan, and many other studies. Similar to the significant cross-cultural differences between Western and Eastern countries, research conducted in Indonesia, one of the Eastern countries, was scarcely found in a peer-reviewed article. The studies given in literature suggested a relationship between Big Five Personality Traits and eating behavior or food choices, for example in a study of 288 students (121 males and 167 females) of *Institut Pertanian Bogor* aged 16−18 years old, the multiple linear regression test results showed that extraversion who enjoys socializing (β = 0.163; *p* = 0.035), openness to experience who has creative and imaginative nature (β = 0.208; *p* = 0.013), and agreeableness who has friendly and environmentally sensitive nature (β = 0.265; *p* = 0.009) tend to choose to eat vegetables for various reasons (Purnamawati and Yuliati, 2016). In another study of 380 students of *Universitas Islam Negeri Suska Riau* aged 18−21 years old, the multiple regression test results showed conscientiousness (rxy = −1.230; *p* = 0.008) and neuroticism (rxy = 0.103; *p* = 0.022) was related to eating behavior (Nelvi, 2016). Additionally, extraversion was related to restrained eating, agreeableness was related to external and emotional eating, and openness to experience was related to emotional, external, and restrained eating (Nelvi, 2016). A study of 195 students (128 males and 67 females) at several universities in South Tangerang linked the Big Five Personality Traits to healthy behavior, one of which was eating behavior, the multiple regression test results showed that personality as a whole did not have a significant influence on healthy behavior, but only openness (*p* = 0.018; *B* = 0.228) and openness among females (*p* = 0.002; *B* = 0.401) had a significant positive effect on healthy behavior based on the regression coefficient (Rahmadian, 2011). Unfortunately, in this study, health behavior (including eating behavior, exercise, smoking, and alcohol consumption) was measured as one, so there was no visible influence from the other four personalities that might only affect certain healthy behaviors (Rahmadian, 2011). As far as we could find, there were no studies that linked physical activity and nutritional status to Big Five Personality Traits in the Indonesian setting and these studies were conducted with a sample size of less than 400 respondents.

For this reason, we conducted this study, especially since evidence related to the association between personality traits, eating habits, physical activity, and nutritional status was scarcely found in a peer-reviewed article in the Indonesian setting and this research was also conducted using secondary data analysis from the Indonesia Family Life Survey (IFLS) with a sample size of tens of thousands, so the results obtained in this study should better describe the existing situation. Therefore, this study aimed to analyze the relationship between Big Five Personality Traits, eating habits, physical activity, and obesity in Indonesia.

## Materials and methods

### Indonesia family life survey

#### Data collection

This study used a quantitative analytic cross sectional design from secondary data analysis of the 2014 Indonesia Family Life Survey (IFLS) or the 5th wave IFLS. IFLS represented about 83% of the Indonesian population (13 out of 26 provinces) in 1993 which was the result of collaboration between the non-profit RAND corporation and Survey Meter (Strauss et al., 2016). IFLS was a continuous longitudinal socio-economic and health survey based on a household sample and had been carried out 5 times, IFLS1 (1993−1994), IFLS2 (1997−1998), IFLS3 (2000), IFLS4 (2007−2008), and IFLS5 (2014−2015) (Strauss et al., 2016).

Since IFLS was a continuous longitudinal survey, the sampling scheme of the first wave will determine the next wave. IFLS used a stratified random sampling technique where the sampling scheme was conducted randomly by the province to maximize population representation and capture the cultural and socio-economic diversity of Indonesia based on the Susenas (National Socioeconomic Survey) in 1993 whose sampling frame was designed by the BPS (Central Bureau of Statistics) based on the 1990 census (Frankenberg and Karoly, 1995). There were 321 EAs (Enumeration Areas) which were randomly selected from each of the 13 provinces with details of the sampling rate applied to each province and resulted in a total EA distribution, and separated by sampling urban EAs and EAs in small provinces to facilitate urban-rural and Javanese-non-Javanese comparisons (Frankenberg and Karoly, 1995). In the selected EA, households were randomly selected based on the 1993 Susenas list, giving a total of 7,730 target sample households, and complete/partial interviews were obtained for 7,039 households, including 22,327 successfully interviewed individuals in IFLS1 (Frankenberg and Karoly, 1995).

In IFLS5, re-contact was established with respondents sampled since IFLS1 in 1993 with a re-contact rate of 92% of original IFLS1 dynasty households (Strauss et al., 2016). IFLS5 was conducted on the same set of households in the previous wave and the same IFLS distribution of 16,204 households and 50,148 individuals were interviewed with 2,662 people who had died since IFLS4 being interviewed with representatives who knew them well (Strauss et al., 2016).

#### Participants

Participants in this study were selected from all people aged ≥ 15 years taken from the IFLS5 dataset. They were chosen, as the information on certain study variables (eating habits and physical activity) was available only to them. From a total of 50,148 participants successfully interviewed on IFLS5, 19,208 participants (38%) were excluded from the study because they were under 15 years old and provided incomplete information regarding the required variables. If a value was missing or answered “don’t know” in one of the studied variables, the respondent would be excluded from the study. Therefore, 30,940 participants (53.2% female) aged 15−101 years in Indonesia were included in this study. The mean age was 37.34 years (*SD* = 14.916).

### Variables and measures

Data was collected using Computer Assisted Personal Interviewing (CAPI) system. This system that uses computers to manage survey questionnaires (all in the Indonesian language) so that interviewers can directly enter data during face-to-face interviews (Strauss et al., 2016). The independent variable studied in this study was personality based on the Big Five Personality Traits. Variables related to socio-demographic characteristics were used as control variables. Whereas, the dependent variables studied were eating habits (recommended and non-recommended foods), physical activity, and obesity based on BMI.

#### Socio-demographic factors

The socio-demographic factors used in this study were age (continuous variable), gender, education (not attending school or attending school), occupation (unemployed, self-employed, or worker), and marital status (never married, married/living together, or divorced/separated). The occupation was defined as the respondent’s primary employment status (Strauss et al., 2016). All these variables were obtained through interviews (Strauss et al., 2016).

#### Big five personality traits

Personality data were obtained through interviews (Strauss et al., 2016). The question used was the Big Five Index 15 (BFI 15), a subset of BFI 44 from John and Srivastava (1999), which consisted of 15 traits representing all five personality dimensions (openness to new experience, conscientiousness, extraversion, agreeableness, neuroticism) with three traits for each personality dimension (Strauss et al., 2016). A short version of BFI, a 15-item instrument, used to measure personality dimensions, was first made available to SOEP (Socio-Economic Panel) in 2005 (Dehne and Schupp, 2007). This instrument was used in many large-scale surveys, such as the German Socio-Economic Panel (GSOEP) (Strauss et al., 2016). The BF1 15 instrument was translated into Indonesian and back into English several times until the English re-translation converged with the BFI 15 English (Strauss et al., 2016). Four lists of the same 15 words were made and the list used was randomly determined in CAPI (Strauss et al., 2016). A five-point ordinal scale was used to represent how well the respondent believes that the trait represents them, 1 = disagree strongly, 2 = disagree a little, 3 = neither agree nor disagree, 4 = agree a little, and 5 = agree strongly (Strauss et al., 2016). In question (R) (reverse), the value was given in reverse from 5 to 1 (John et al., 1991). Scores on each personality were summed based on the five personality dimensions.

We tested the validity and reliability of the BFI 15 scale using SPSS version 20 with 30,940 respondents based on the number of samples that would be used in this study. Item validity was seen through the value of each item with Pearson product-moment. Items were considered valid if the significance value was *p* < 0.05. Based on these criteria, all item validity test results were considered valid. Meanwhile, the reliability test was performed using an internal consistency approach which was analyzed using the Cronbach Alpha formula. The determination of the high and low-reliability coefficient was based on Guilford’s reliability coefficient classification (Sugiyono, 2011). The result of the reliability test showed that the alpha coefficient value was 0.402. Based on Guilford’s reliability coefficient classification, a coefficient value of more than 0.40 to 0.70 means the variable has sufficient reliability category. Therefore, the scale was reliable enough to be used.

#### Eating habits

Eating habits data were obtained through interviews (Strauss et al., 2016). The foods were representative of foods rich in iron and vitamin A, two micronutrients considered deficient among the Indonesian population, and also include fast foods, fried foods, and sweet snacks to get at some unhealthy eating habits (Strauss et al., 2016). Eating habits were defined as the consumption frequency (number of days) by food type (17 types) in the past week which could be answered by never or between 1 to 7 days which were then classified as not consuming (answered never) and consuming (answered between 1 to 7 days) in this study. The 17 types of food were sweet potatoes, eggs, fish, meat (beef, chicken, pork, etc.), dairy, green leafy vegetables, banana, papaya, carrot, mango, *sambal*, rice, including five non-recommended foods were instant noodle, fast food, soft drink (coca cola, sprite, etc.), fried snacks (*tempe, tahu, bakwan*, etc.), and sweet snacks (*wajik, geplak*, donuts, wafers, chocolate, etc.).

#### Physical activity

Physical activity data were obtained through interviews with a set of questions that were taken from an international survey on physical activities about the types and times of physical activities carried out in all parts of life (at work, home, and exercise) for at least 10 min continuously in the last 7 days (Strauss et al., 2016). The questionnaire consisted of similar questions as GPAQ but was simpler and translated into Indonesian, therefore the calculation and classification of physical activity were based on GPAQ (Global Physical Activity Questionnaire). However, because in the questionnaire activity duration was only asked for in a time frame, we chose the closest number from that time frame so that METs-minutes/week could be calculated. Respondents who answered < 30 min were counted as 15 min, ≥30 min counted as 60 min, <4 h counted as 3 h (180 min), and ≥4 h counted as 5 h (300 min). In this study, the classification of high and moderate levels of physical activity based on reference values (GPAQ Analysis Guide Version 2 by World Health Organization [WHO], 2005) were grouped into one in high and moderate. Therefore, the level of physical activity was then classified into high and moderate, and low.

#### Obesity

To obtain BMI, the required height data were obtained through measurement using a Seca plastic height board, model 213, measured to the nearest millimeter, meanwhile, body weight was measured using a Camry model EB1003 digital scale, measured to the nearest tenth (one decimal point) of a kilogram (Strauss et al., 2016). Obesity of respondents aged ≥ 19 years was determined by BMI and < 19 years was determined by BMI-for-age (BMI/A) using WHO Anthro plus software. The cut-off point used refers to the regulation of the Indonesian Ministry of Health which was based on the WHO reference 2007 for children aged 5 to 18 years (Regulation of the Indonesian Ministry of Health number 2 of 2020 Indonesian Ministry of Health [IMH], 2020) and based on the FAO/WHO provisions where for the benefit of Indonesia, the cut-off point was modified based on clinical experience and research results in several developing countries to determine the BMI of individuals aged > 19 years (Regulation of the Indonesian Ministry of Health number 41 of 2014 Indonesian Ministry of Health [IMH], 2014). In this study, the classification of overweight and obesity based on reference values were grouped into one as obese. Therefore, obesity was categorized into: not obese for BMI: ≤25.0 and BMI/A: ≤+1 SD and obese for BMI: >25.0 and BMI/A: >+1 SD.

### Data analysis

The data that had been collected were tabulated and then tested for statistical correlation with binary logistic regression. First, the bivariate test was conducted between personality (each personality dimension based on the Big Five Personality Traits) and nutrition-related variables (eating habits, physical activity, and obesity), then continued with a multivariate test controlled by socio-demographic factors (sex, age, marital status, education, and occupation) conducted on significant variables in the bivariate test to examine the independent effect of personality. The covariates were predetermined based on previous studies that showed a confounding effect on the relationship between the studied variables (Terracciano et al., 2009; Kye and Park, 2012; Mõttus et al., 2013; Tiainen et al., 2013; Sutin and Terracciano, 2015a,b; Sutin et al., 2016; Gustavsen and Rickertsen, 2019; Pfeiler and Egloff, 2020). To examine whether the association between personality traits and nutrition-related variables varied by sex or age group (adolescent, adult, elderly), we also tested this interaction, controlling for socio-demographic factors. Data analysis was carried out using Statistical Package for the Social Sciences (SPSS version 20). A significance level of 95% and an error rate of α = 5% (0.05). Significant relationships between the tested variables were determined if the *p* < 0.05.

### Ethical considerations

This study using secondary data analysis was granted ethical clearance by the Airlangga University Faculty of Dental Medicine Health Research Ethical Clearance Commission.

## Results

### Descriptive statistics

The total sample in this study included 30,940 respondents. All characteristics of study participants are presented in Tables 1, 2.

**TABLE 1:** Characteristic of study participants.

Characteristics	Frequency (*n*)	Percentages (%)
**Gender**		
Male	14,473	46.8
Female	16,467	53.2
**Age**		
Adolescent (15−18 years)	2,868	9.3
Adult (19−65 years)	26,553	85.8
Elderly (>65 years)	1,519	4.9
**Marital status**		
Married/living together	22,459	72.6
Divorced/separated	2,378	7.7
Never married	6,103	19.7
**Education**		
Not attending school	1,191	3.8
Attending school	29,749	96.2
**Occupation**		
Unemployed	8,407	27.2
Self-employed	8,548	27.6
Worker	13,985	45.2
**Eating habits Sweet potatoes**		
Not consuming	20,061	64.8
Consuming	10,879	35.2
**Eggs**		
Not consuming	5,342	17.3
Consuming	25,598	82.7
**Fish**		
Not consuming	6,097	19.7
Consuming	24,843	80.3
Meat (beef, chicken, pork, etc.)		
Not consuming	11,559	37.4
Consuming	19,381	62.6
**Dairy**		
Not consuming	21,201	68.5
Consuming	9,739	31.5
Green leafy vegetables		
Not consuming	3,691	11.9
Consuming	27,249	88.1
**Banana**		
Not consuming	15,007	48.5
Consuming	15,933	51.5
**Papaya**		
Not consuming	22,933	74.1
Consuming	8,007	25.9
**Carrot**		
Not consuming	19,996	64.6
Consuming	10,944	35.4
**Mango**		
Not consuming	20,401	65.9
Consuming	10,539	34.1
**Instant noodle**		
Not consuming	10,555	34.1
Consuming	20,385	65.9
**Fast food**		
Not consuming	27,506	88.9
Consuming	3,434	11.1
**Soft drink (Coca cola, sprite, etc.)**		
Not consuming	24,922	80.5
Consuming	6,018	19.5
***Sambal***		
Not consuming	5,877	19.0
Consuming	25,063	81.0
**Fried snacks (*tempe, tahu, bakwan*, etc.)**		
Not consuming	10,777	34.8
Consuming	20,163	65.2
**Rice**		
Not consuming	53	0.2
Consuming	30,887	99.8
**Sweet snacks (*wajik, geplak*, donuts, wafers, chocolate, etc.)**		
Not consuming	14,642	47.3
Consuming	16,298	52.7
**Physical Activity**		
Low	13,872	44.8
Moderate and high	17,068	55.2
**Obesity**		
Not obesity	21,207	68.5
Obesity	9,733	31.5

**TABLE 2:** Characteristic of study participants based on Big Five Personality Traits.

Big Five Personality Traits	Mean ± SD (min-max)
	
Openness	11.11 ± 2.006(3−15)
Conscientiousness	11.45 ± 1.656(3−15)
Extraversion	10.33 ± 1.998(3−15)
Agreeableness	11.70 ± 1.533(3−15)
Neuroticism	8.04 ± 1.996(3−15)

Of the 30,940 respondents aged 15−101 years, most were women (53.2%), adults (85.8%), married/living together (72.6%), attending school (96.2%), and working (45.2%), and the mean age was 37.34 years (*SD* = 14.916). Most of them consumed animal protein in the form of eggs, fish, and meat, rice as carbohydrate sources, green leafy vegetables, banana, *sambal*, and three non-recommended foods: fried snacks, instant noodles, and sweet snacks. Meanwhile, most of them did not consume dairy as animal protein sources, sweet potatoes as carbohydrate sources, papaya, mango, carrot, and two non-recommended foods: fast food and soft drink. Respondents mainly consumed rice (99.8%) and did not consume fast food (88.9%). Most of the respondents had moderate and high levels of physical activity (55.2%) which was consistent with their nutritional status, most of them were classified as not obese (68.5%).

Table 2 shows that among the five dimensions of the Big Five Personality Traits, the dimension with the highest average score is agreeableness (11.70 ± 1.533). Meanwhile, the dimension with the lowest average score is neuroticism (8.04 ± 1.996).

### Regression results

To examine the relationship between personality (each personality dimension based on the Big Five Personality Traits) and nutrition-related variables (eating habits, physical activity, and obesity), statistical tests were performed using binary logistic regression due to the large sample size, the data were not normally distributed, and assumption of proportional odds was not met. Supplementary Tables 1, 2 present the regression results on the bivariate test for each variable tested in this study.

From the eating habits of 17 types of food tested, including five non-recommended foods: instant noodles, fast food, soft drink (coca cola, sprite, etc.), fried snacks (*tempe, tahu, bakwan*, etc.), and sweet snacks (*wajik, geplak*, donuts, wafers, chocolate, etc.), Supplementary Table 1 shows that openness and extraversion had a significant association with eating habits of all food types except rice. Conscientiousness was associated with eating all food types except mango, rice, and two non-recommended foods (instant noodle and fried snacks). Agreeableness was associated with eating fish, green leafy vegetables, fruits (banana, papaya, mango), carrot, and four of the five non-recommended foods (fast food, soft drink, fried snacks, and sweet snacks). Meanwhile, neuroticism was unrelated to eating eggs, fish, *sambal*, and rice. Therefore, only rice consumption did not have a significant association with all dimensions of Big Five Personality Traits. In addition, the eating habit of green leafy vegetables, bananas, papaya, carrot, fast food, soft drink, and sweets snacks were associated with all dimensions of the Big Five Personality Traits.

There was also a statistically significant association between the level of physical activity with openness (*p* = 0.007), conscientiousness (*p* < 0.001), and agreeableness (*p* = 0.008). Conscientiousness was 1.070 times more likely to have more high physical activities than agreeableness (OR = 1.020; 95% CI = 1.005−1.035) and openness (OR = 1.016; 95% CI = 1.004−1.027). There was also a statistically significant association between obesity and conscientiousness (*p* < 0.001), extraversion (*p* < 0.001), and neuroticism (*p* < 0.001). Extraversion was 1.093 times more likely to be obese than conscientiousness (OR = 1.052; 95% CI = 1.037−1.068). Meanwhile, neuroticism has a protective effect on obesity (OR = 0.977; 95% CI = 0.965−0.989). Next step, a multivariate test controlled with socio-demographic factors (sex, age, marital status, education, and occupation) was conducted on the significant variables in the bivariate test to examine the independent effect of personality.

Supplementary Table 2 shows that there was still a statistically significant association between all food types tested with openness even after controlling for socio-demographic factors, which meant that openness was associated with both recommended and non-recommended foods. All food types showed a positive association, which meant respondents with a higher openness were more likely to consume the tested food type than those with a lower openness. The highest possibility was found in green leafy vegetables (OR = 1.107; CI = 1.088−1.126) and the lowest was in fried snacks (OR = 1.019; CI = 1.007−1.031). In addition, only sweet snacks where all control variables had a significant correlation with the association between openness and eating habits according to the food type.

Openness was also positively associated in adults for all food types tested, except eggs, meat, dairy, green leafy vegetables, fast food, and sweet snacks did not vary by age group. The association was stronger in the elderly between openness with sweet potatoes (OR = 1.067; CI = 1.020−1.117), fish (OR = 1.112; CI = 1.058−1.168), and instant noodle (OR = 1.070; CI = 1.022−1.121) than in adults ([OR = 1.030; CI = 1.017−1.043]; [OR = 1.053; CI = 1.037−1.070], [OR = 1.056; CI = 1.042−1.070]), but no association was found in adolescents and the rest food types were not associated in adolescents and the elderly. In addition, the relationship between openness and all food types tested did not vary by sex, except fried snacks in males (OR = 1.026; CI = 1.008−1.044), and no association was found in females.

Supplementary Table 3 shows that after controlling for socio-demographic factors, only three non-recommended foods (fast food, soft drink, and sweet snacks) had no statistically significant association, while the others were positively associated with conscientiousness. The highest possibility was found in the green leafy vegetables (OR = 1.101; CI = 1.079−1.124) and the lowest was in sweet potatoes (OR = 1.018; CI = 1.003−1.032). Only meat where all control variables had a significant correlation with the association between conscientiousness and eating habits according to the food type.

Conscientiousness was also found to be positively related to all food types tested in adults, except green leafy vegetables, fast food, soft drink, and sweet snacks did not vary by age group. The results also showed that conscientiousness was related to fish (OR = 1.056; CI = 1.008−1.106), meat (OR = 0.949; CI = 0.909−0.991), banana (OR = 1.049; CI = 1.007−1.093), and *sambal* (OR = 0.939; CI = 0.892−0.988) in adolescents and eggs (OR = 1.073; CI = 1.008−1.042), dairy (OR = 1.143; CI = 1.065−1.226), and papaya (OR = 1.075; CI = 1.006−1.148) in the elderly. The association between conscientiousness and all food types tested did not vary by sex, except dairy (OR = 1.051; CI = 1.028−1.074) and fast food (OR = 1.046; CI = 1.011−1.082) in males and soft drink in females (OR = 0.968; CI = 0.942−0.994).

Supplementary Table 4 shows that after controlling for socio-demographic factors, only fish had no statistically significant association, while the others were positively associated with extraversion, which meant that extraversion was associated with both recommended and non-recommended foods. The highest probability was found in fast food (OR = 1.081; CI = 1.062−1.101) and the lowest was in sweet potatoes, mango, and instant noodle (OR = 1.023; CI = 1.011−1.036). Only sweet snacks where all control variables had a significant correlation with the association between extraversion and eating habits according to the food type.

Extraversion was also positively associated in adults for all food types tested, except meat, dairy, and sweet snacks, which did not vary by age group. The relationship between extraversion and all food types tested did not vary by sex, except sweet potatoes (OR = 1.034; CI = 1.018−1.051), carrot (OR = 1.043; CI = 1.027−1.060), and mango (OR = 1.029; CI = 1.013−1.046) in females, also, no association was found in males. However, the results showed that this relationship was stronger in females for eggs (OR = 1.065; CI = 1.043−1.087), meat (OR = 1.081; CI = 1.063−1.098), dairy (OR = 1.058; CI = 1.040−1.076), green leafy vegetables (OR = 1.041; CI = 1.016−1.066), instant noodle (OR = 1.026; CI = 1.009−1.043), fast food (OR = 1.092; CI = 1.066−1.118), soft drink (OR = 1.067; CI = 1.043−1.091), *sambal* (OR = 1.068; CI = 1.047−1.089), fried snacks (OR = 1.026; CI = 1.009−1.042), and sweet snacks (OR = 1.044; CI = 1.028−1.061) than in males. This showed that higher extraversion in females was more likely to consume all kinds of healthy and unhealthy foods than males.

Supplementary Table 5 shows that after controlling for socio-demographic factors, only carrots, fast food, fried snacks, and sweet snacks had no statistically significant association with agreeableness. All food types that had a significant association were positively associated with agreeableness and only soft drink (non-recommended food) was negatively associated. This meant respondents with higher agreeableness had a greater probability of not consuming soft drinks compared to those with lower agreeableness. The highest possibility was found in green leafy vegetables (OR = 1.041; CI = 1.018−1.064).

A negative association between agreeableness and soft drink consumption was found especially in adolescents (OR = 0.942; CI = 0.896−0.990) and in adults (OR = 0.977; CI = 0.957−0.997), but this association did not vary by sex. This negative association was also found in the consumption of fried snacks in males (OR = 0.976; CI = 0.954−0.999) and in adults (OR = 0.981; CI = 0.964−0.997), and sweet snacks in adults (OR = 0.981; CI = 0.966−0.997). In addition, agreeableness was associated in adults for green leafy vegetables (OR = 1.028; CI = 1.003−1.054), banana (OR = 1.027; CI = 1.011−1.044), papaya (OR = 1.024; CI = 1.006−1.043), and mango (OR = 1.018; CI = 1.001−1.035).

Supplementary Table 6 shows that after controlling for socio-demographic factors, only sweet potatoes, banana, papaya, mango, and fast food had no statistically significant association with neuroticism. Four non-recommended foods: instant noodle, soft drink, fried snacks, and sweet snacks were positively associated with neuroticism, while four recommended foods: meat, dairy, green leafy vegetables, and carrot were negatively associated. Fried snacks have the highest OR (OR = 1.021; CI = 1.009−1.033) and the lowest was green leafy vegetables (OR = 0.942; CI = 0.925−0.958). This meant that respondents with higher neuroticism increased their consumption of fried snacks by 1.021 times and decreased their consumption of green leafy vegetables by 0.942 times compared to those with lower neuroticism. Only meat and sweet snacks where all control variables had a significant correlation with the association between neuroticism and eating habits according to the food type.

Neuroticism was also associated in adults with all those four recommended foods (negative association) and four non-recommended foods (positive association) with the same association. The association between neuroticism and sweet snacks was stronger in the elderly (OR = 1.081; CI = 1.025−1.140) than in adults (OR = 1.016; CI = 1.004−1.029), but no association was found in adolescents. Additionally, neuroticism was also associated with soft drinks (OR = 1.024; CI = 1.001−1.047) and sweet snacks (OR = 1.018; CI = 1.001−1.034) in females, and fried snacks in males (OR = 1.029; CI = 1.011−1.048). The relationship between neuroticism with dairy, green leafy vegetables, and instant noodles did not vary by sex.

Table 3 shows that after controlling for socio-demographic factors, only agreeableness had no statistically significant association with physical activity. Openness (*p* = 0.010) and conscientiousness (*p* < 0.001) were positively associated with physical activity. Respondents with higher conscientiousness had a 1.045 times higher probability of engaging in more intense physical activity than those with lower conscientiousness, while openness had a lower probability of 1.015 times. In addition, all gender, marital status, education, and occupation were correlated with this association.

**TABLE 3:** Multivariate analysis between Big Five Personality Traits with physical activity and control variables.

Variables	Physical Activity			
	
	*P*-Value	OR	95% CI	
			
			Lower	Upper
Openness	0.010	1.015*	1.004	1.027
Age	0.075	1.002	1.000	1.004
Gender				
Male	<0.001	1.218*	1.159	1.280
**Female (referent)**				
**Marital status**				
Married/living together	<0.001	1.325*	1.236	1.420
Divorced/separated	0.023	1.148*	1.019	1.292
**Never married (referent)**				
**Education**				
Not attending school	<0.001	1.273*	1.122	1.445
Attending school (referent)				
**Occupation**				
Unemployed	<0.001	0.702*	0.662	0.743
Self-employed	<0.001	1.182*	1.116	1.251
**Worker (referent)**				
Conscientiousness	<0.001	1.045*	1.030	1.059
Age	0.186	1.001	0.999	1.003
**Gender**				
Male	<0.001	1.221*	1.162	1.283
**Female (referent)**				
**Marital status**				
Married/living together	<0.001	1.298*	1.210	1.391
Divorced/separated	0.045	1.129*	1.003	1.271
**Never married (referent)**				
**Education**				
Not attending school	<0.001	1.278*	1.127	1.450
Attending school (referent)				
**Occupation**				
Unemployed	<0.001	0.710*	0.670	0.752
Self-employed	<0.001	1.186*	1.121	1.256
**Worker (referent)**				
Agreeableness	0.286	1.008	0.993	1.023
Age	0.123	1.002	1.000	1.004
**Gender**				
Male	<0.001	1.223*	1.164	1.285
**Female (referent)**				
**Marital status**				
Married/living together	<0.001	1.321*	1.232	1.415
Divorced/separated	0.029	1.141*	1.013	1.284
**Never married (referent)**				
**Education**				
Not attending school	<0.001	1.261*	1.112	1.431
Attending school (referent)				
Occupation				
Unemployed	<0.001	0.700*	0.661	0.742
Self-employed	<0.001	1.183*	1.117	1.252
**Worker (referent)**				

*Significant at p < 0.05 using binary logistic regression test.

The relationship between physical activity and agreeableness did not vary by sex or age group. Meanwhile, physical activity was found to be associated with conscientiousness in adolescents (OR = 1.059; CI = 1.016−1.103) and adults (OR = 1.042; CI = 1.026−1.058), but not in the elderly. This association did not vary by sex. In addition, openness was associated with physical activity only in females (OR = 1.018; CI = 1.002-1.034) and the elderly (OR = 1.065; CI = 1.017-1.115).

Table 4 shows that there was still a statistically significant association between conscientiousness (*p* = 0.002), extraversion (*p* < 0.001), and neuroticism (*p* < 0.001) with obesity even after controlling for socio-demographic factors. Extraversion and conscientiousness were positively associated with obesity, which meant respondents with higher extraversion were 1.079 times more likely and conscientiousness was 1.025 times more likely to be obese compared to respondents with lower scores. Meanwhile, neuroticism had a negative association or protective effect on obesity (OR = 0.966; CI = 0.953−0.978). In addition, all gender, marital status, age, education, and occupation (except self-employed) were correlated with this association.

**TABLE 4:** Multivariate analysis between Big Five Personality Traits with obesity and control variables.

Variables	Obesity			
	
	*P*-Value	OR	95% CI	
			
			Lower	Upper
Conscientiousness	0.002	1.025*	1.009	1.041
Age	<0.001	1.011*	1.009	1.013
**Gender**				
Male	<0.001	0.429*	0.406	0.453
**Female (referent)**				
**Marital status**				
Married/living together	<0.001	2.500*	2.291	2.729
Divorced/separated	<0.001	1.593*	1.392	1.824
**Never married (referent)**				
**Education**				
Not attending school	<0.001	0.461*	0.400	0.532
Attending school (referent)				
**Occupation**				
Unemployed	0.004	0.911*	0.854	0.971
Self-employed	0.272	1.035	0.974	1.099
**Worker (referent)**				
Extraversion	<0.001	1.079*	1.065	1.092
Age	<0.001	1.012*	1.010	1.014
**Gender**				
Male	<0.001	0.444*	0.420	0.469
**Female (referent)**				
**Marital status**				
Married/living together	<0.001	2.546*	2.333	2.779
Divorced/separated	<0.001	1.637*	1.429	1.875
**Never married (referent)**				
**Education**				
Not attending school	<0.001	0.480*	0.416	0.553
**Attending school (referent)**				
**Occupation**				
Unemployed	0.008	0.916*	0.859	0.977
Self-employed	0.468	1.023	0.962	1.087
Worker (referent)				
Neuroticism	<0.001	0.966*	0.953	0.978
Age	<0.001	1.011*	1.009	1.013
**Gender**				
Male	<0.001	0.421*	0.398	0.445
**Female (referent)**				
**Marital status**				
Married/living together	<0.001	2.519*	2.308	2.748
Divorced/separated	<0.001	1.601*	1.398	1.833
**Never married (referent)**				
**Education**				
Not attending school	<0.001	0.459*	0.399	0.529
Attending school (referent)				
**Occupation**				
Unemployed	0.004	0.910*	0.853	0.970
Self-employed	0.302	1.033	0.972	1.097
**Worker (referent)**				

*Significant at p < 0.05 using binary logistic regression test.

Conscientiousness was associated with obesity only in females (OR = 1.031; CI = 1.010−1.051) and in adults (OR = 1.027; CI = 1.010−1.044). Neuroticism was associated with obesity only in males (OR = 0.935; CI = 0.916−0.955) and in adults (OR = 0.957; CI = 0.944−0.970). Meanwhile, extraversion was associated with obesity in adolescents (OR = 1.069; CI = 1.012−1.128) and in adults (OR = 1.078; CI = 1.064−1.092), but not in the elderly. The association between obesity and extraversion also appeared to be stronger in females (OR = 1.083; CI = 1.066−1.101) than in males (OR = 1.074; CI = 1.053−1.096). This showed that women who have higher extraversion were more likely to be overweight or obese.

## Discussion

### Big five personality traits and eating habits

Eating habits were found related to personality, so personality was assessed as contributing to certain eating patterns (Mõttus et al., 2013; Sutin and Terracciano, 2015b). Based on data on the consumption of 17 types of food (including five types of non-recommended foods: instant noodles, fast food, soft drink, fried snacks, and sweet snacks) and personality based on the Big Five Personality Traits, also after controlling for socio-demographic factors (age, gender, education, occupation, and marital status), the following results were obtained. This study showed that rice was not associated with all dimensions of personality, which could be because the staple food of the Indonesians is rice and very few people do not eat it.

This study found openness was positively associated (especially in adults), both with recommended and non-recommended foods, which was positively associated with eating all food types tested, and the most likely positive association was with green leafy vegetables and the lowest was with fried snacks only a slight difference. This may indicate the nature of openness to accepting various types of food, both healthy and unhealthy. This study also found a positive association between openness and fried snacks in men. This might be because men tended to have higher openness scores than women (Magee and Heaven, 2011; Armon et al., 2013; Intiful et al., 2019).

In previous studies, openness was associated with a Mediterranean-style diet and avoidance of sweet-based forms of diet (Mõttus et al., 2013), moreover, openness was found to be the most consistent and strongest result when associated with eating healthy foods (Mõttus et al., 2013; Tiainen et al., 2013). Openness was positively associated with eating plant-based food and fish (Pfeiler and Egloff, 2020), fruits and vegetables (Tiainen et al., 2013; Keller and Siegrist, 2015; Conner et al., 2017), and was negatively associated with eating meat (Keller and Siegrist, 2015; Pfeiler and Egloff, 2020), sugary drinks (Keller and Siegrist, 2015), and potato chips (Conner et al., 2017), however, other studies showed that openness was associated with increased consumption of wine (Gustavsen and Rickertsen, 2019) and non-recommended products (Gacek et al., 2021). The different results can be explained by the characteristics of openness that are more open to new experiences, as well as greater curiosity and exploration, making them more open to change so that they are more open to habits that are considered sustainable, for example, it is easier to adopt new adaptive eating patterns and overcome taste aversions (Mõttus et al., 2013; Conner et al., 2017), including consuming healthier foods such as fruits and vegetables, but if healthy eating habit has been habituated or perceived to be more familiar, it may no longer match the characteristics of higher openness (Mõttus et al., 2013). The different results were also possible due to the variation in the characteristics of the studied sample which may lead to different behavioral tendencies considering that openness may adopt healthy or unhealthy eating behaviors.

This study found that conscientiousness was positively associated with eating recommended foods (especially in adults and except mango, which was not associated), with the highest probability of consuming green leafy vegetables (did not vary by sex or age group). Conscientiousness was not associated with all non-recommended foods: fast food, soft drink, instant noodle, fried snacks, and sweet snacks. This study also found a negative association between conscientiousness and meat in adolescents and soft drinks in women and a positive association between dairy and fast food in men.

Consistent with the previous studies, conscientiousness tends to choose healthy foods and foods recommended in dietary guidelines (Keller and Siegrist, 2015), as in the study by Mõttus et al. (2013) higher conscientiousness was associated with a tendency to have a healthier diet. In the study by Gacek et al. (2021), there was an increase in eating most recommended products and a decrease in eating most non-recommended products, and in the study by Ibigbami (2012), conscientiousness was found to be negatively associated with alcohol consumption, which was similar to previous studies, where it seemed that conscientiousness had a holistic effect not only on alcohol use but on all aspects of their wellbeing. Conscientiousness was also found to be positively associated with eating plant-based food and fish (Pfeiler and Egloff, 2020), fruits (Keller and Siegrist, 2015), and the combined daily consumption of vegetables and fruits compared to lower conscientiousness (Conner et al., 2017). Conscientiousness was negatively associated with eating meat (Keller and Siegrist, 2015; Pfeiler and Egloff, 2020), sugary drinks, sweet and savory foods (Keller and Siegrist, 2015), and foods containing preservatives (among young women) (Jaworski and Rozenek, 2016). Additionally, increasing conscientiousness also reduces the expected frequency of beer consumption (Gustavsen and Rickertsen, 2019). Meanwhile, lower conscientiousness tends to perceive their eating habits as more unhealthy and have poor eating habits (Lawler, 2018). This is due to conscientiousness tends to be more careful in choosing the food consumed (Keller and Siegrist, 2015; Nelvi, 2016). Furthermore, it is also due to the level of discipline and orderliness that affects adherence to healthy diets (such as Mediterranean diet) and avoidance of unhealthy foods (such as sweet foods) (Mõttus et al., 2013).

In this study, extraversion was positively associated with eating all types of food tested (except fish, which was not associated), which meant a positive association with eating both recommended and non-recommended foods (especially higher in women than men), with the highest probability of consuming fast food. This can be supported by previous studies, women scored higher on extraversion than men (Magee and Heaven, 2011; Armon et al., 2013; Purnamawati and Yuliati, 2016).

Previous studies showed that extraversion was negatively associated with eating carbohydrate-based food (Pfeiler and Egloff, 2020) and was positively associated with eating meat (Keller and Siegrist, 2015; Pfeiler and Egloff, 2020), plant-based food, and fish (Pfeiler and Egloff, 2020), fruits and vegetables (Purnamawati and Yuliati, 2016; Conner et al., 2017), whole-grain bread (Jaworski and Rozenek, 2016), sugary drinks, sweet and savory foods (Keller and Siegrist, 2015), alcohol (Ibigbami, 2012; Kye and Park, 2012), wine (Gustavsen and Rickertsen, 2019), and Mediterranean diet (Mõttus et al., 2013). Other studies on young women also found that individuals with higher extraversion tend to choose foods that are considered to have a good impact on health (Jaworski and Rozenek, 2016). Based on those results, it can be seen that extraversion tends to increase consumption of both recommended and non-recommended foods. This was also seen in the study by Gacek et al. (2021) who suggested that more extroverted individuals tend to be more assertive, optimistic, open, sensation-seeking, and happy to socialize may increase consumption of most recommended products, but consumption of non-recommended products decreased with red meat and lard, while increased with sweets and alcohol. This may be due to extraversion who prefer to socialize and is more attached to social networks (Friedman et al., 2010) so they need energy from gathering with other people. Therefore, they regularly attend social situations and eat more often with other people, but the food selection is limited to the menu provided (Tiainen et al., 2013) so it is possible to frequently consume non-recommended foods which can have a negative impact on health (Keller and Siegrist, 2015). In this study, perhaps the biggest tendency was meeting other people in restaurants selling fast food, as this study found that the highest probability was fast food consumption.

In this study, agreeableness was positively associated with eating several recommended foods: fish, fruits (banana, papaya, mango, especially in adults), and most likely green leafy vegetables (especially in adults), and negatively associated with eating non-recommended foods: soft drink (especially in adolescents and adults), fried snacks in men and adults, and sweet snacks in adults. The rest of the food types were unrelated. The results of this study were in line with previous studies, higher agreeableness was associated with a healthier diet (Mõttus et al., 2013). In previous studies, agreeableness was found to be positively associated with eating plant-based food and fish (Pfeiler and Egloff, 2020) and fruit (in women) (Tiainen et al., 2013). Agreeableness was negatively associated with eating carbohydrate-based food (Pfeiler and Egloff, 2020), meat (Keller and Siegrist, 2015), and frequency of wine and beer consumption (Gustavsen and Rickertsen, 2019). However, other studies showed that the association between agreeableness and eating habits appears to be rather weak and inconsistent across studies (Pfeiler and Egloff, 2020). Such agreeableness was found to be associated with increased consumption of non-recommended products (Gacek et al., 2021), and infrequent avoidance of salty foods (among young women) (Jaworski and Rozenek, 2016), and poor or unhealthy diet patterns (Kye and Park, 2012). It may be that agreeableness consumes less meat because ethical concerns based on high altruism and sympathy also apply to animals (Keller and Siegrist, 2015), or agreeableness was significantly negatively associated with alcohol consumption because individuals with high agreeableness are quite sociable and naturally have good and respectful natures, so they are easy to adopt other forms of coping strategies that could reduce the tendency to use or abuse alcohol (Ibigbami, 2012), or other traits such as obedience make them more likely to adhere to rules such as dietary guidelines, but other traits that tend to be easy to trust and have a high tolerance of others, happy to help, kind and gentle, make them easily influenced by their social environment which may force them to follow unhealthy dietary practices (Rahmadian, 2011; Kye and Park, 2012). These might contradict and confuse them, but as adults, they might begin to realize the benefits of dietary guidelines, therefore in this study the healthy eating habit was found particularly in higher agreeableness adults.

This study found that neuroticism was positively associated with eating almost all non-recommended foods (especially in adults): instant noodles, soft drinks, sweet snacks, and the highest probability was eating fried snacks (fast food was unrelated). Neuroticism was also positively associated with soft drinks and sweet snacks in women and fried snacks in men. In addition, neuroticism was negatively associated with eating several recommended foods (especially in adults): meat, dairy, carrot, and the highest probability of green leafy vegetable consumption, the rest of the food types were unrelated.

Consistent with this finding, neuroticism was found to be associated with poorer diet quality (Tiainen et al., 2013). In previous studies, neuroticism was positively associated with eating carbohydrate-based food and meat (Pfeiler and Egloff, 2020), sweet and savory foods (Keller and Siegrist, 2015), soft drinks (for women) (Tiainen et al., 2013), French fries (for men) (Conner et al., 2017), and non-recommended products (including sweets) (Gacek et al., 2021). Neuroticism was negatively associated with plant-based food and fish (Pfeiler and Egloff, 2020), vegetables (in women) (Tiainen et al., 2013), recommended whole grain cereals (Gacek et al., 2021), and the Mediterranean diet (Mõttus et al., 2013) which dietary recommendations encourage eating healthy foods. This can happen because individuals with high neuroticism have low self-esteem, high sensitivity, and are emotionally unstable (Gacek et al., 2021), which causes their mood to fluctuate often, also easily feel upset and disturbed, which is then overcome by eating because they react to eating behavior as a form of reassurance over negative emotions (Elfhag and Morey, 2008; Nelvi, 2016), thereby ignoring healthy food choices. It can be worse, especially in productive age or adulthood, where stress levels are higher and they are more prone to depression. Meanwhile, individuals with emotional stability (low levels of neuroticism) tend to choose foods considered to have a good impact on health (Jaworski and Rozenek, 2016).

### Big five personality traits and physical activity

In this study, only openness, conscientiousness, and agreeableness were associated with physical activity. However, after controlling for age, gender, education, occupation, and marital status, only openness and conscientiousness remained associated with physical activity, but age did not seem to moderate this association. Meanwhile, the association between agreeableness and physical activity was weakened by these socio-demographic factors so that it no longer had a statistically significant association.

This study found that extraversion was not associated and conscientiousness was positively associated with physical activity. Slight differences were found in previous findings that high extraversion and conscientiousness (as well as low neuroticism) were found to be associated with higher or more active levels of physical activity (Rhodes, 2006; Rhodes and Smith, 2006; Allen and Laborde, 2014; Stephan et al., 2014b; Mõttus et al., 2016). Individuals with higher extraversion and conscientiousness tend to have more energy (Terracciano et al., 2013) and an active nature is theorized as a key aspect of extraversion and conscientiousness that represents their tendency to be busy and energetic (Rhodes, 2006). Thus, physical activity can be used as an activity in accordance with the energy capacity they need (Stephan et al., 2014b). Individuals with low extraversion may be less likely to engage in physical activity because they find it less enjoyable and have a lower level of control over it than people with higher extraversion (Rhodes, 2006). Meanwhile, individuals who lack order and self-discipline (low conscientiousness) are likely to have more difficulty maintaining their physical activity plans (Rhodes, 2006). Extraversion and conscientiousness also largely have a positive association with activity variability within individuals (Mõttus et al., 2016). The difference in results on extraversion found in this study may be due to the fact that Indonesians have very low levels of physical activity compared to other countries (Althoff et al., 2017), so although individuals with high extraversion have more energy and like to gather with other people, this is directed toward activities other than physical activity and their social environment also do not support physical activity due to the sedentary habits of the community. Therefore, extraversion was not associated with physical activity in this study.

This study also found that individuals with higher conscientiousness in adolescents and adults tended to be more active, but there was no association in the elderly. This might be because age was positively correlated with a decreased physical activity where children were more active than adults (Miles, 2007) and physical activity steadily decreased in the later stages of life (Wilson and Dishman, 2015). However, age was also related to personality such as conscientiousness which tended to increase with age (Brummett et al., 2006). These perhaps became very contradictory in old age, thus negating the relationship between conscientiousness and physical activity in the elderly.

This study found that openness was positively associated with physical activity (especially in women and the elderly), which was consistent with previous findings. Stephan et al. (2014a) showed that not only extraversion, but openness was also associated with the possibility of being involved in various physical, cognitive, and social activities, and also found a consistent pattern in two different Western societies (United States and France), that there were intrinsic tendencies toward extraversion and openness to having a more active lifestyle. These characteristics are perhaps more dominant in women and older people, as they tend to prefer to gather, talk, and do activities together, including physical activities. Openness characteristics which have a tendency to new ideas and experiences and also prefer variety are characteristics of individuals who are open and can lead to active involvement in various types of activities (Stephan et al., 2014a) thus enabling individuals to have a higher level of physical activity because physical activity can encourage a tendency to explore and seek new and varied experiences (Stephan et al., 2014b).

In this study, agreeableness was found to be unrelated to physical activity. This result was consistent with other studies that also found no association between agreeableness and physical activity, perhaps suggesting that the tendency to trust and help others is unrelated to motivation and involvement in physical activity (Courneya and Hellsten, 1998; Sutin et al., 2016; Intiful et al., 2019). However, in the study by Stephan et al. (2014a), agreeableness showed a positive association with variations in the type of activity in one of the samples tested. Physical activity also encourages a tendency to be exposed to various bodily, emotional, and social experiences which can also produce social interactions that may contribute to the maintenance of a prosaic orientation that characterizes agreeableness (Stephan et al., 2014b).

In this study, neuroticism was unrelated to physical activity, but in previous studies, neuroticism was negatively associated with physical activity in one of the samples tested in the studies by Stephan et al. (2014a,b). Overall, neuroticism seems to be negatively associated with physical activity but has little impact (Rhodes and Smith, 2006). Theoretically, neuroticism with a tendency to feel insecure and jealous may look to others for motivation to behave (Rhodes, 2006) for example in physical activity. Feelings of worry about appearance, feelings of obligation, and feelings of guilt if they do not exercise become central to their motivation for physical activity (Ingledew and Markland, 2008), but due to their low self-control which is reflected by impulsivity (Elfhag and Morey, 2008), it is difficult for them to maintain the intention and schedule in exercising. This may have a contradictory impact on neuroticism and therefore neuroticism was unrelated to physical activity.

The results of this study were slightly different from previous studies. This may be due to variations and differences in the characteristics of the respondents studied since a study related to the association between Big Five Personality Traits and physical activity has never been studied before in Indonesians.

### Big five personality traits and obesity

In this study, both before and after being controlled by socio-demographic factors, conscientiousness, extraversion, and neuroticism were associated with obesity, it seemed that all control variables moderated this association except working as self-employed. Meanwhile, openness and agreeableness were not associated with obesity.

The positive association between conscientiousness and obesity (especially in women and adults) found in this study was not unexpected. Compared to other dimensions, low conscientiousness and high neuroticism had the strongest associations with BMI and obesity (Sutin and Terracciano, 2015b). But based on a meta-analysis by Jokela et al. (2013), only conscientiousness was consistently associated with obesity. Inconsistent results were found for other dimensions in previous studies. Previous studies showed that conscientiousness was negatively associated with nutritional status, which meant that low conscientiousness was associated with higher nutritional status (obesity) in adult samples (Magee and Heaven, 2011; Sutin et al., 2011; Sutin and Terracciano, 2015b; Pfeiler and Egloff, 2020) and older people (Mõttus et al., 2013). Conscientiousness was also found to be negatively associated with several measures of body weight (Armon et al., 2013). Conscientiousness was associated with healthier body weight, possibly because high conscientiousness was characterized as conscientious, efficient, orderly, obedient, organized, and disciplined individuals, so they were more likely to have behaviors that support a healthy weight or maintain the desired body shape, such as eating habits, following a meal plan, eating at the same time every day, and exercising (Brummett et al., 2006; Terracciano et al., 2009; Sutin et al., 2011; Sutin and Terracciano, 2015b). Meanwhile, low conscientiousness had difficulties in self-control, so they tend to overeat, lack physical activity, and had poor eating habits such as chaotic eating schedules, which can then lead to obesity (Magee and Heaven, 2011; Sutin and Terracciano, 2015b). Although conscientiousness was associated with healthier eating habits and more physically active found in this study, the different results in association with nutritional status may be due to other mechanisms linking this association. For example, an imbalance in total energy intake and energy expenditure that was not investigated in this study or other possibilities due to physiological factors such as health conditions or history of disease. Moreover, several previous studies found an association between personality dimensions and several physiological and genetic mechanisms that can then affect body composition (Kakizaki et al., 2008), such as the finding of low conscientiousness associated with higher circulating levels of leptin (Sutin et al., 2013) and FTO genetic variant had little effect on the relationship between personality traits with underweight and overweight (Terracciano et al., 2009). Therefore, future studies may further investigate other mechanisms that might underlie this association.

This study found that neuroticism was negatively associated with obesity (especially in men and adults). However, previous studies found inconsistent findings such as a negative association between neuroticism and nutritional status (Kakizaki et al., 2008) as well as a positive association (Sutin et al., 2011; Pfeiler and Egloff, 2020). Individuals with low conscientiousness or high neuroticism were prone to obesity (Sutin et al., 2011). High neuroticism was characterized by individuals who tend to have emotional vulnerability, fear, anxiety, and depression related to eating behavior for comfort due to negative emotions, poor eating habits, avoidance of physical activity, and impulsiveness associated with emotional eating can lead to obesity (Elfhag and Morey, 2008; Magee and Heaven, 2011). This can be seen in low self-discipline (conscientiousness) and impulsiveness (neuroticism) which reflect poor individual self-control (Elfhag and Morey, 2008). Moreover, this impulsivity trait was the strongest predictor of nutritional status which was found in many studies (Terracciano et al., 2009). However, Terracciano et al. (2009) found that lean people scored higher on neuroticism, but scored lower on impulsivity. The inconsistent results on neuroticism and obesity might be explained by the nature of their susceptibility to depression, especially in productive age or adulthood. Depression can trigger individuals to eat more or less, so it was strongly associated with emotion and nutritional status (Yilmaz and Köse, 2020). This can lead individuals to have a more or less nutritional status. As in this study, although neuroticism was found to be positively associated with eating non-recommended foods and negatively associated with eating recommended foods, but neuroticism was negatively associated with obesity, this might be related to their susceptibility to depression so that it affected the nutritional status that tended to be not obese.

In this study, extraversion was positively associated with obesity (especially in adolescents, adults, and higher in women). This finding was consistent with other studies that found a positive association between extraversion and nutritional status (Magee and Heaven, 2011; Sutin et al., 2011), but another study found that extraversion was negatively associated with BMI among women but not among men (Sutin and Terracciano, 2015b). Brummett et al. (2006) showed that men with higher extraversion had a higher BMI explained by extraversion traits that are adventurous, fun-seeking, sociable, and gregarious, which may lead them to consume more high-calorie foods and/or consume more alcohol. Armon et al. (2013) offered an alternative explanation that the tendency of individuals with high positive moods to be more careless, feel less vulnerable to unwanted health conditions, and adopt unhealthy behaviors that lead to overweight. Although in this study extraversion was associated with eating recommended and non-recommended foods (especially higher in women), which reflected their lack of attention to the food they consumed, which then may have an impact on obesity, in another study, extraversion was associated with greater physical activity (Hoyt et al., 2009), which means they actually have the potential to be able to maintain a healthy weight.

Our findings could be supported by findings from previous studies that obesity affects British and Indonesian women more than men (Mihardja and Soetrisno, 2012), and women tended to consume more sweet foods such as candy and chocolate (Conner et al., 2017). Another study also found that men had higher quality food intake and eating habits than women (Puspadewi, 2014), but other studies found the opposite (Mõttus et al., 2013). In addition, the increase in BMI occurs mainly in young and middle adults, then after passing the age of 60 years, body weight decreases rapidly and returns to a value comparable to that of young adults (Sutin et al., 2011; Suiraoka, 2012; Chaudhuri and Bandopadhyay, 2014).

This study found no association between obesity with openness and agreeableness. Previous studies showed inconsistent findings, which suggested that nutritional status was unrelated to agreeableness (Pfeiler and Egloff, 2020) and openness (Sutin and Terracciano, 2015a; Pfeiler and Egloff, 2020), positively associated with openness (Sutin et al., 2011), also negatively (Sutin et al., 2011; Sutin and Terracciano, 2015a) and positively associated with agreeableness (Magee and Heaven, 2011). Openness was characterized by an open and curios nature may be easier to adopt a new adaptive eating pattern, but perhaps if the eating habit has been habituated or perceived to be more familiar, it may no longer match the characteristics of higher openness (Mõttus et al., 2013). Meanwhile, agreeableness was characterized by individuals who are warm, empathetic and need the consideration of others, which may lead to contradictory eating behaviors that can then affect body weight (Armon et al., 2013). This may be what causes the different findings in various studies or even the lack of association between nutritional status with openness and agreeableness.

Differences in results with previous studies may be due to variations in the sample tested and methodological differences such as the composition of the Big Five Personality Trait questions used differently from previous studies. Due to inconsistent findings across many studies, it is difficult to draw conclusions. Thus, further study is needed to describe a more specific mechanism regarding the association between personality and nutritional status.

### What can we learn?

In this study, openness was found to be positively associated with both recommended and non-recommended foods (especially in adults), positively associated with physical activity (especially in women and the elderly), but not associated with obesity. This can be explained by the fact that even though they were more active, the nature of individuals with high openness tend to like variety and be open to new experiences and ideas, which allows them to more easily follow new healthy or unhealthy dietary guidelines, which leads to openness did not determine nutritional status. Therefore, individuals with high openness should be directed to adaptive behavior patterns that come with a lot of variety so that they can meet their needs for variety and their interest in new experiences, such as food menus or healthy types of diets that regularly changed various physical activities. Meanwhile, low openness maybe enjoys a more fixed schedule, not too varied, and a selection of menus and activities that are more familiar to them due to their more conservative and conventional tendencies.

Conscientiousness was positively associated with eating almost all recommended foods (especially in adults) and not associated with all non-recommended foods in this study. Moreover, conscientiousness was positively associated with physical activity (especially in adolescents and adults), but also positively associated with obesity (especially in women and adults), which was an unexpected result. This may be because in this study conscientiousness was not associated with eating non-recommended foods, so they could have high or low levels of consumption, although conscientiousness increased the possibility of consuming recommended foods and physical activity, which then impacted obesity, or there may be another mechanism underlying this relationship (such as their knowledge of healthy foods which was not examined in this study). Therefore, individuals with high conscientiousness can be directed to adaptive behavior with clear and organized guidelines and schedules so that it will be easier to adopt because it is in line with their conscientious, disciplined, organized, and obedient nature. Meanwhile, low conscientiousness may need behavioral strategies, implementation intentions, and behavior change goals such as increasing physical activity (Rhodes, 2006).

This study found that extraversion was positively associated with eating recommended and non-recommended foods (especially higher in women), was not associated with physical activity and was positively associated with obesity (especially in adolescents, adults, and higher in women). This was possible because food choices are disregarded by individuals with high extraversion due to their tendency to like to gather and even eat with other people more often, which may limit their food choices (Tiainen et al., 2013), in addition to being more careless and inattentive their attention to unwanted health conditions (Armon et al., 2013) which then has an impact on obesity. Therefore, individuals with high extraversion may be more suited to group activities and activities that require a lot of energy as it suits their sociable and energetic nature to make it easier for them to adopt adaptive behavior. Meanwhile, low extraversion may be able to take advantage of behavior change strategies that do not require much energy and do not involve many people, such as exercising at home.

Agreeableness was positively associated with eating several recommended foods and negatively associated with eating non-recommended foods (especially in adults). However, agreeableness was unrelated to physical activity and obesity. This may be because the nature of individuals with high agreeableness who like to help and easily trust others has no effect on their motivation and involvement in physical activities (Courneya and Hellsten, 1998; Sutin et al., 2016; Intiful et al., 2019). In addition, their tendency to obey and trust others leads them to be easily influenced by their social environment which can lead them to adopt healthy or unhealthy dietary guidelines depending on the individual who directs them or individuals they trust, causing contradictory eating behaviors and agreeableness did not determine nutritional status. Therefore, individuals with high agreeableness can be directed to adaptive behavior by involving the people around them, especially those they trust, so that they can adopt the behavior more easily. Meanwhile, low agreeableness may benefit from behavior change strategies related to independence and avoid the involvement of others, as they tend to be suspicious and critical of others, such as preparing their own healthy meals.

This study found that neuroticism was positively associated with eating four of the five non-recommended foods and negatively associated with four recommended foods (especially in adults), and the rest was unrelated. Moreover, neuroticism was found to be unrelated to physical activity and negatively associated with obesity (especially in men and adults). This may be because individuals with high neuroticism tend to be unstable, emotional, and impulsive, which indicates their low self-control, so they find it harder to maintain the intention to do physical activity, but in addition, they may engage in physical activity because they feel insecure or see others as motivation to exercise (Rhodes, 2006), so neuroticism was unrelated to physical activity in this study. Additionally, individuals with high neuroticism susceptibility to stress may cause them to tend to have low intakes in this study which might protect them from obesity even though they had poor quality food choices. However, if poor eating habits are taken frequently and continuously, it will have a negative impact on their nutritional and health status. Therefore, individuals with higher neuroticism may be able to involve friends or family with lower neuroticism in making behavioral changes, such as exercising regularly (Rhodes, 2006), and can benefit from menu planning and regular meal times.

### Strengths and limitations

The strengths of this study included a large study with over 30,000 respondents so that the results can be more representative of the study population. Our statistical analysis also included control variables to obtain the independent effect of personality on nutrition-related variables. In addition, since evidence related to the association between personality traits, eating habits, physical activity, and nutritional status was scarcely found in peer-reviewed articles in the Indonesian setting, this study is, therefore, a relatively new and pioneering study linking personality and nutrition in an Indonesian setting. Limitations included limited data quality control and the availability of personality and nutrition-related variables, as we used secondary data. Not all samples were used in this study due to data completeness, but among the excluded respondents, there was no indication that they had a BMI that tended to be higher or lower, so there was no systematic bias. The physical activity duration data processing also used an approach method because IFLS5 had only time frame data, which might lead to overestimation or underestimation. Another limitation, measuring obesity using BMI has a weakness because it is unable to distinguish between body fat and fat-free mass (Johansson et al., 2009). The absence of body composition in assessing obesity leads to a risk of bias, therefore more precise measurements of obesity are recommended, such as fat mass and waist circumference (Johansson et al., 2009). Lastly, this study was a cross-sectional study, so it was not possible to draw conclusions about causality.

## Conclusion

After controlling for socio-demographic variables, openness was positively associated with recommended and non-recommended foods, and physical activity, and not associated with obesity. Conscientiousness was positively associated with almost all recommended food types, physical activity, and obesity, and not associated with all non-recommended foods. Extraversion was positively associated with recommended and non-recommended foods and obesity, and not associated with physical activity. Agreeableness was positively associated with several recommended foods, negatively associated with one non-recommended food, and not associated with physical activity and obesity. Neuroticism was positively associated with almost all non-recommended foods, negatively associated with four recommended foods and obesity, was also not associated with physical activity. Behavior change can take into account individual characteristics such as personalized strategies with personality because the adaptation of personality characteristics can be modified even though the personality tends to be persistent. This strategy can be used to improve nutritional status or public health because personality, particularly conscientiousness, was found to be consistent with previous studies across western and eastern countries that conscientiousness had a relationship with eating habits and physical activity. Additionally, higher extraversion (especially in women) was found to be more likely to be careless in food choices and more prone to obesity. Therefore, the understanding of personality needs to be improved so that health and nutrition improvement interventions are more effective and allow for sustainability as the interventions or programs are most suited to the needs of individual preferences. Programs to prevent obesity, improve physical activity, and healthy eating habits should be tailored to accommodate individuals with low conscientiousness personalities especially women with higher extraversion. This information can be useful not only to health professionals, but also to teachers, psychologists, and the community who target the promotion of healthy living or the prevention of nutrition and health problems.

## Data availability statement

Publicly available datasets were analyzed in this study. This data can be found here: https://www.rand.org/well-being/social-and-behavioral-policy/data/FLS/IFLS/ifls5.html

## Author contributions

GP and TM conceived the project and research question. GP conducted the data analyses and wrote the original draft of the manuscript. TM and MR reviewed and edited the manuscript. TM was responsible for funding the publication of the manuscript. All authors contributed to data processing and read and agreed to the published version of the manuscript.
