# Supplementary material for: The relationship between Big Five Personality Traits, eating habits, physical activity, and obesity in Indonesia based on analysis of the 5th wave Indonesia Family Life Survey (2014)
Source: Front Psychol. 2022 Aug 4;13:881436. doi: 10.3389/fpsyg.2022.881436 (PMC9387636; doi:10.3389/fpsyg.2022.881436)
Supplement: Supplementary file 1 [file Data_Sheet_1.docx]

Supplementary File

The Relationship between Big Five Personality Traits, Eating Habits, Physical Activity, and Obesity in Indonesia Based on Analysis of the 5th Wave Indonesia Family Life Survey (2014)

Greena Pristyna^1^, Trias Mahmudiono^1^*, Mahmud A. Rifqi^1^, and Diah Indriani^2^

^1^Department of Nutrition, Faculty of Public Health, Airlangga University, Surabaya, Indonesia

^2^Department of Biostatistics and Demography, Faculty of Public Health, Airlangga University, Surabaya, Indonesia

*** Correspondence:**Trias Mahmudiono
trias-m@fkm.unair.ac.id

Table 1. Bivariate Analysis between the Five Dimensions of Big Five Personality Traits and Eating Habits, Physical Activity, Obesity

| **Variables**  **Big Five**  **Personality Traits** | | | **Eating Habits** | | | | | | | | |
| --- | --- | --- | --- | --- | --- | --- | --- | --- | --- | --- | --- |
|  |  |  | **Sweet potatoes** | **Eggs** | **Fish** | **Meat (beef, chicken, pork, etc.)** | **Dairy** | **Green leafy vegetables** | **Banana** | **Papaya** | **Carrot** |
| **Openness** | p-value | | 0.001 | <0.001 | <0.001 | <0.001 | <0.001 | <0.001 | <0.001 | <0.001 | <0.001 |
|  | OR | | 1.020* | 1.107* | 1.060* | 1.105* | 1.112* | 1.103* | 1.043* | 1.029* | 1.060* |
|  | 95% CI | Lower | 1.008 | 1.091 | 1.046 | 1.093 | 1.098 | 1.085 | 1.031 | 1.016 | 1.047 |
|  |  | Upper | 1.032 | 1.123 | 1.075 | 1.118 | 1.125 | 1.121 | 1.054 | 1.043 | 1.072 |
| **Conscientiousness** | p-value | | <0.001 | <0.001 | <0.001 | <0.001 | 0.043 | <0.001 | <0.001 | <0.001 | <0.001 |
|  | OR | | 1.041* | 1.043* | 1.072* | 1.038* | 1.015* | 1.119* | 1.051* | 1.046* | 1.057* |
|  | 95% CI | Lower | 1.026 | 1.025 | 1.054 | 1.024 | 1.001 | 1.097 | 1.037 | 1.030 | 1.042 |
|  |  | Upper | 1.055 | 1.062 | 1.090 | 1.053 | 1.030 | 1.141 | 1.065 | 1.062 | 1.073 |
| **Extraversion** | p-value | | <0.001 | <0.001 | 0.029 | <0.001 | <0.001 | <0.001 | <0.001 | <0.001 | <0.001 |
|  | OR | | 1.022* | 1.077* | 1.016* | 1.075* | 1.052* | 1.044* | 1.029* | 1.031* | 1.039* |
|  | 95% CI | Lower | 1.010 | 1.061 | 1.002 | 1.062 | 1.039 | 1.026 | 1.017 | 1.018 | 1.027 |
|  |  | Upper | 1,034 | 1.093 | 1.030 | 1.087 | 1.065 | 1.062 | 1.040 | 1.044 | 1.051 |
| **Agreeableness** | p-value | | 0.244 | 0.743 | <0.001 | 0.613 | 0.351 | <0.001 | <0.001 | 0.001 | 0.028 |
|  | OR | | 1.009 | 1.003 | 1.034* | 1.004 | 1.007 | 1.045* | 1.036* | 1.030* | 1.017* |
|  | 95% CI | Lower | 0.994 | 0.984 | 1.015 | 0.989 | 0.992 | 1.022 | 1.021 | 1.013 | 1.002 |
|  |  | Upper | 1.025 | 1.023 | 1.053 | 1.019 | 1.023 | 1.068 | 1.051 | 1.047 | 1.003 |
| **Neuroticism** | p-value | | 0.034 | 0.624 | 0.212 | <0.001 | 0.001 | <0.001 | <0.001 | 0.002 | <0.001 |
|  | OR | | 0.987* | 0.996 | 0.991 | 0.978* | 0.979* | 0.944* | 0.980* | 0.980* | 0.979* |
|  | 95% CI | Lower | 0.976 | 0.982 | 0.977 | 0.967 | 0.967 | 0.928 | 0.969 | 0.968 | 0.967 |
|  |  | Upper | 0.999 | 1.011 | 1.005 | 0.989 | 0.991 | 0.961 | 0.991 | 0.993 | 0.990 |

*Significant at p <0.05 using binary logistic regression test

Table 1. Bivariate Analysis between the Five Dimensions of Big Five Personality Traits and Eating Habits, Physical Activity, Obesity (cont’d)

| **Variables**  **Big Five**  **Personality Traits** | | | **Eating Habits** | | | | | | | | **Physical Activity** | **Obesity** |
| --- | --- | --- | --- | --- | --- | --- | --- | --- | --- | --- | --- | --- |
|  |  |  | **Mango** | **Instant noodle** | **Fast food** | **Soft drink (Coca cola, sprite, etc.)** | ***Sambal*** | **Fried snacks (*tempe, tahu, bakwan*, etc.)** | **Rice** | **Sweet snacks (*wajik, geplak*, donuts, wafers, chocolate, etc.)** |  |  |
| **Openness** | p-value | | 0.002 | <0.001 | <0.001 | <0.001 | <0.001 | <0.001 | 0.342 | <0.001 | 0.007 | 0.806 |
|  | OR | | 1.019* | 1.079* | 1.127* | 1.118* | 1.076* | 1.033* | 1.065 | 1.099* | 1.016* | 1.002 |
|  | 95% CI | Lower | 1.007 | 1.066 | 1.106 | 1.102 | 1.061 | 1.021 | 0.936 | 1.087 | 1.004 | 0.990 |
|  |  | Upper | 1.031 | 1.092 | 1.148 | 1.135 | 1.091 | 1.045 | 1.212 | 1.112 | 1.027 | 1.014 |
| **Conscientiousness** | p-value | | 0.631 | 0.229 | 0.011 | 0.001 | <0.001 | 0.380 | 0.469 | 0.001 | <0.001 | <0.001 |
|  | OR | | 1.003 | 0.991 | 0.973* | 0.971* | 1.045* | 0.994 | 1.060 | 0.977* | 1.070* | 1.052* |
|  | 95% CI | Lower | 0.989 | 0.977 | 0.952 | 0.954 | 1.027 | 0.980 | 0.905 | 0.964 | 1.055 | 1.037 |
|  |  | Upper | 1.018 | 1.006 | 0.994 | 0.987 | 1.062 | 1.008 | 1.241 | 0.990 | 1.084 | 1.068 |
| **Extraversion** | p-value | | <0.001 | <0.001 | <0.001 | <0.001 | <0.001 | <0.001 | 0.815 | <0.001 | 0.086 | <0.001 |
|  | OR | | 1.027* | 1.036* | 1.098* | 1.042* | 1.073* | 1.028* | 1.016 | 1.050* | 1.010 | 1.093* |
|  | 95% CI | Lower | 1.015 | 1.024 | 1.079 | 1.028 | 1.057 | 1.016 | 0.888 | 1.038 | 0.999 | 1.080 |
|  |  | Upper | 1.039 | 1.048 | 1.118 | 1.057 | 1.088 | 1.041 | 1.163 | 1.062 | 1.021 | 1.107 |
| **Agreeableness** | p-value | | 0.015 | 0.497 | 0.004 | <0.001 | 0.946 | 0.009 | 0.403 | <0.001 | 0.008 | 0.318 |
|  | OR | | 1.019* | 1.005 | 0.967* | 0.963* | 1.001 | 0.980* | 1.076 | 0.967* | 1.020* | 1.008 |
|  | 95% CI | Lower | 1.004 | 0.990 | 0.945 | 0.946 | 0.982 | 0.965 | 0.906 | 0.953 | 1.005 | 0.992 |
|  |  | Upper | 1.035 | 1.021 | 0.989 | 0.981 | 1.019 | 0.995 | 1.278 | 0.981 | 1.035 | 1.024 |
| **Neuroticism** | p-value | | 0.044 | <0.001 | 0.003 | 0.016 | 0.131 | <0.001 | 0.672 | <0.001 | 0.130 | <0.001 |
|  | OR | | 1.012* | 1.039* | 1.027* | 1.017* | 1.011 | 1.025* | 1.030 | 1.046* | 0.991 | 0.977* |
|  | 95% CI | Lower | 1.000 | 1.027 | 1.009 | 1.003 | 0.997 | 1.013 | 0.899 | 1.035 | 0.980 | 0.965 |
|  |  | Upper | 1.024 | 1.052 | 1.045 | 1.032 | 1.026 | 1.037 | 1.180 | 1.058 | 1.003 | 0.989 |

*Significant at p <0.05 using binary logistic regression test

Table 2. Multivariate Analysis between Openness with Eating Habits and Control Variables

| Variables | Eating Habits | | | | | | | | | | | | | |
| --- | --- | --- | --- | --- | --- | --- | --- | --- | --- | --- | --- | --- | --- | --- |
|  | Sweet potatoes | | | |  | Eggs | | | |  | Fish | | | |
|  | p-value | OR | 95% CI | |  | p-value | OR | 95% CI | |  | p-value | OR | 95% CI | |
|  |  |  | Lower | Upper |  |  |  | Lower | Upper |  |  |  | Lower | Upper |
| Openness | <0.001 | 1.037* | 1.024 | 1.049 |  | <0.001 | 1.082* | 1.066 | 1.098 |  | <0.001 | 1.056* | 1.041 | 1.071 |
| Age | <0.001 | 1.019* | 1.017 | 1.021 |  | <0.001 | 0.985* | 0.983 | 0.988 |  | 0.164 | 0.998 | 0.996 | 1.001 |
| Gender |  |  |  |  |  |  |  |  |  |  |  |  |  |  |
| Male | <0.001 | 0.875* | 0.831 | 0.921 |  | 0.013 | 0.920* | 0.862 | 0.982 |  | 0.840 | 0.994 | 0.934 | 1.057 |
| Female (referent) |  |  |  |  |  |  |  |  |  |  |  |  |  |  |
| Marital status |  |  |  |  |  |  |  |  |  |  |  |  |  |  |
| Married/living together | 0.002 | 1.125* | 1.044 | 1.213 |  | <0.001 | 1.245* | 1.132 | 1.369 |  | <0.001 | 1.369* | 1.258 | 1.490 |
| Divorced/separated | 0.211 | 0.924 | 0.816 | 1.046 |  | 0.722 | 1.027 | 0.885 | 1.193 |  | 0.311 | 1.076 | 0.934 | 1.241 |
| Never married (referent) |  |  |  |  |  |  |  |  |  |  |  |  |  |  |
| Education |  |  |  |  |  |  |  |  |  |  |  |  |  |  |
| Not attending school | <0.001 | 0.660* | 0.580 | 0.751 |  | <0.001 | 0.463* | 0.406 | 0.528 |  | <0.001 | 0.733* | 0.673 | 0.845 |
| Attending school (referent) |  |  |  |  |  |  |  |  |  |  |  |  |  |  |
| Occupation |  |  |  |  |  |  |  |  |  |  |  |  |  |  |
| Unemployed | <0.001 | 0.881* | 0.829 | 0.937 |  | 0.028 | 0.917* | 0.849 | 0.991 |  | <0.001 | 0.787* | 0.734 | 0.844 |
| Self-employed | 0.171 | 1.041 | 0.983 | 1.103 |  | 0.013 | 0.911* | 0.847 | 0.980 |  | 0.094 | 1.064 | 0.990 | 1.143 |
| Worker (referent) |  |  |  |  |  |  |  |  |  |  |  |  |  |  |

*Significant at p <0.05 using binary logistic regression test

Table 2. Multivariate Analysis between Openness with Eating Habits and Control Variables (cont’d)

| Variables | Eating Habits | | | | | | | | | | | | | |
| --- | --- | --- | --- | --- | --- | --- | --- | --- | --- | --- | --- | --- | --- | --- |
|  | Meat (beef, chicken, pork, etc) | | | |  | Dairy | | | |  | Green leafy vegetables | | | |
|  | p-value | OR | 95% CI | |  | p-value | OR | 95% CI | |  | p-value | OR | 95% CI | |
|  |  |  | Lower | Upper |  |  |  | Lower | Upper |  |  |  | Lower | Upper |
| Openness | <0.001 | 1.085* | 1.072 | 1.098 |  | <0.001 | 1.094* | 1.080 | 1.108 |  | <0.001 | 1.107* | 1.088 | 1.126 |
| Age | 0.373 | 0.999 | 0.997 | 1.001 |  | 0.737 | 1.000 | 0.998 | 1.003 |  | 0.265 | 0.998 | 0.995 | 1.001 |
| Gender |  |  |  |  |  |  |  |  |  |  |  |  |  |  |
| Male | 0.057 | 1.051 | 0.999 | 1.106 |  | <0.001 | 1.137* | 1.079 | 1.199 |  | <0.001 | 0.788* | 0.730 | 0.850 |
| Female (referent) |  |  |  |  |  |  |  |  |  |  |  |  |  |  |
| Marital status |  |  |  |  |  |  |  |  |  |  |  |  |  |  |
| Married/living together | <0.001 | 0.772* | 0.717 | 0.831 |  | <0.001 | 0.625* | 0.582 | 0.672 |  | <0.001 | 1.664* | 1.503 | 1.843 |
| Divorced/separated | <0.001 | 0.631* | 0.558 | 0.712 |  | <0.001 | 0.706* | 0.621 | 0.803 |  | 0.038 | 1.200* | 1.010 | 1.424 |
| Never married (referent) |  |  |  |  |  |  |  |  |  |  |  |  |  |  |
| Education |  |  |  |  |  |  |  |  |  |  |  |  |  |  |
| Not attending school | <0.001 | 0.373* | 0.328 | 0.424 |  | <0.001 | 0.382* | 0.320 | 0.455 |  | <0.001 | 0.669* | 0.567 | 0.789 |
| Attending school (referent) |  |  |  |  |  |  |  |  |  |  |  |  |  |  |
| Occupation |  |  |  |  |  |  |  |  |  |  |  |  |  |  |
| Unemployed | <0.001 | 0.882* | 0.831 | 0.937 |  | 0.181 | 0.958 | 0.901 | 1.020 |  | <0.001 | 0.825* | 0.756 | 0.899 |
| Self-employed | 0.021 | 0.934* | 0.882 | 0.990 |  | 0.035 | 0.937* | 0.881 | 0.995 |  | 0.313 | 0.956 | 0.875 | 1.044 |
| Worker (referent) |  |  |  |  |  |  |  |  |  |  |  |  |  |  |

*Significant at p <0.05 using binary logistic regression test

Table 2. Multivariate Analysis between Openness with Eating Habits and Control Variables (cont’d)

| Variables | Eating Habits | | | | | | | | | | | | | |
| --- | --- | --- | --- | --- | --- | --- | --- | --- | --- | --- | --- | --- | --- | --- |
|  | Banana | | | |  | Papaya | | | |  | Carrot | | | |
|  | p-value | OR | 95% CI | |  | p-value | OR | 95% CI | |  | p-value | OR | 95% CI | |
|  |  |  | Lower | Upper |  |  |  | Lower | Upper |  |  |  | Lower | Upper |
| Openness | <0.001 | 1.061* | 1.049 | 1.073 |  | <0.001 | 1.047* | 1.033 | 1.061 |  | <0.001 | 1.077* | 1.064 | 1.090 |
| Age | <0.001 | 1.025* | 1.023 | 1.027 |  | <0.001 | 1.016* | 1.014 | 1.019 |  | <0.001 | 1.013* | 1.011 | 1.015 |
| Gender |  |  |  |  |  |  |  |  |  |  |  |  |  |  |
| Male | 0.369 | 0.978 | 0.930 | 1.027 |  | <0.001 | 0.795* | 0.751 | 0.840 |  | <0.001 | 0.692* | 0.657 | 0.729 |
| Female (referent) |  |  |  |  |  |  |  |  |  |  |  |  |  |  |
| Marital status |  |  |  |  |  |  |  |  |  |  |  |  |  |  |
| Married/living together | 0.204 | 0.956 | 0.891 | 1.025 |  | 0.007 | 1.121* | 1.032 | 1.217 |  | 0.005 | 1.110* | 1.031 | 1.195 |
| Divorced/separated | 0.017 | 0.864* | 0.767 | 0.974 |  | 0.259 | 0.925 | 0.808 | 1.059 |  | 0.921 | 1.006 | 0.889 | 1.139 |
| Never married (referent) |  |  |  |  |  |  |  |  |  |  |  |  |  |  |
| Education |  |  |  |  |  |  |  |  |  |  |  |  |  |  |
| Not attending school | <0.001 | 0.582* | 0.514 | 0.659 |  | <0.001 | 0.651* | 0.564 | 0.752 |  | <0.001 | 0.384* | 0.332 | 0.445 |
| Attending school (referent) |  |  |  |  |  |  |  |  |  |  |  |  |  |  |
| Occupation |  |  |  |  |  |  |  |  |  |  |  |  |  |  |
| Unemployed | 0.001 | 0.908* | 0.857 | 0.962 |  | 0.245 | 0.961 | 0.900 | 1.027 |  | 0.004 | 1.092* | 1.028 | 1.159 |
| Self-employed | 0.002 | 1.093* | 1.033 | 1.156 |  | 0.887 | 0.995 | 0.935 | 1.060 |  | 0.001 | 0.907* | 0.855 | 0.962 |
| Worker (referent) |  |  |  |  |  |  |  |  |  |  |  |  |  |  |

*Significant at p <0.05 using binary logistic regression test

Table 2. Multivariate Analysis between Openness with Eating Habits and Control Variables (cont’d)

| Variables | Eating Habits | | | | | | | | | | | | | |
| --- | --- | --- | --- | --- | --- | --- | --- | --- | --- | --- | --- | --- | --- | --- |
|  | Mango | | | |  | Instant noodle | | | |  | Fast food | | | |
|  | p-value | OR | 95% CI | |  | p-value | OR | 95% CI | |  | p-value | OR | 95% CI | |
|  |  |  | Lower | Upper |  |  |  | Lower | Upper |  |  |  | Lower | Upper |
| Openness | 0.001 | 1.020* | 1.008 | 1.032 |  | <0.001 | 1.046* | 1.034 | 1.059 |  | <0.001 | 1.104* | 1.082 | 1.125 |
| Age | 0.213 | 0.999 | 0.997 | 1.001 |  | <0.001 | 0.967* | 0.965 | 0.969 |  | <0.001 | 0.977* | 0.973 | 0.980 |
| Gender |  |  |  |  |  |  |  |  |  |  |  |  |  |  |
| Male | <0.001 | 0.905* | 0.860 | 0.953 |  | 0.003 | 1.082* | 1.027 | 1.141 |  | <0.001 | 0.771* | 0.713 | 0.833 |
| Female (referent) |  |  |  |  |  |  |  |  |  |  |  |  |  |  |
| Marital status |  |  |  |  |  |  |  |  |  |  |  |  |  |  |
| Married/living together | 0.795 | 0.990 | 0.921 | 1.065 |  | <0.001 | 1.290* | 1.195 | 1.394 |  | <0.001 | 0.701* | 0.634 | 0.775 |
| Divorced/separated | 0.212 | 1.082 | 0.956 | 1.223 |  | <0.001 | 1.306* | 1.152 | 1.481 |  | 0.058 | 0.824 | 0.675 | 1.006 |
| Never married (referent) |  |  |  |  |  |  |  |  |  |  |  |  |  |  |
| Education |  |  |  |  |  |  |  |  |  |  |  |  |  |  |
| Not attending school | 0.122 | 0.902 | 0.791 | 1.028 |  | <0.001 | 0.772* | 0.680 | 0.875 |  | <0.001 | 0.286* | 0.189 | 0.432 |
| Attending school (referent) |  |  |  |  |  |  |  |  |  |  |  |  |  |  |
| Occupation |  |  |  |  |  |  |  |  |  |  |  |  |  |  |
| Unemployed | 0.774 | 0.991 | 0.933 | 1.053 |  | 0.292 | 0.967 | 0.908 | 1.029 |  | 0.246 | 0.949 | 0.869 | 1.037 |
| Self-employed | 0.003 | 1.094* | 1.032 | 1.160 |  | 0.758 | 0.991 | 0.934 | 1.051 |  | 0.191 | 0.938 | 0.853 | 1.032 |
| Worker (referent) |  |  |  |  |  |  |  |  |  |  |  |  |  |  |

*Significant at p <0.05 using binary logistic regression test

Table 2. Multivariate Analysis between Openness with Eating Habits and Control Variables (cont’d)

| Variables | Eating Habits | | | | | | | | | | | | | |
| --- | --- | --- | --- | --- | --- | --- | --- | --- | --- | --- | --- | --- | --- | --- |
|  | Soft drink (Coca cola, sprite, etc) | | | |  | *Sambal* | | | |  | Fried snacks (*tempe, tahu, bakwan*, etc) | | | |
|  | p-value | OR | 95% CI | |  | p-value | OR | 95% CI | |  | p-value | OR | 95% CI | |
|  |  |  | Lower | Upper |  |  |  | Lower | Upper |  |  |  | Lower | Upper |
| Openness | <0.001 | 1.071* | 1.054 | 1.087 |  | <0.001 | 1.055* | 1.040 | 1.070 |  | 0.002 | 1.019* | 1.007 | 1.031 |
| Age | <0.001 | 0.973* | 0.970 | 0.976 |  | <0.001 | 0.987* | 0.984 | 0.989 |  | <0.001 | 0.995* | 0.993 | 0.997 |
| Gender |  |  |  |  |  |  |  |  |  |  |  |  |  |  |
| Male | <0.001 | 2.026* | 1.901 | 2.159 |  | 0.484 | 0.978 | 0.918 | 1.041 |  | 0.003 | 1.081* | 1.027 | 1.138 |
| Female (referent) |  |  |  |  |  |  |  |  |  |  |  |  |  |  |
| Marital status |  |  |  |  |  |  |  |  |  |  |  |  |  |  |
| Married/living together | <0.001 | 0.647* | 0.596 | 0.703 |  | <0.001 | 1.185* | 1.083 | 1.297 |  | 0.006 | 0.903* | 0.839 | 0.972 |
| Divorced/separated | 0.028 | 0.833* | 0.707 | 0.980 |  | 0.680 | 0.970 | 0.840 | 1.120 |  | 0.334 | 0.941 | 0.832 | 1.064 |
| Never married (referent) |  |  |  |  |  |  |  |  |  |  |  |  |  |  |
| Education |  |  |  |  |  |  |  |  |  |  |  |  |  |  |
| Not attending school | <0.001 | 0.622* | 0.489 | 0.792 |  | <0.001 | 0.717* | 0.626 | 0.821 |  | <0.001 | 0.584* | 0.517 | 0.661 |
| Attending school (referent) |  |  |  |  |  |  |  |  |  |  |  |  |  |  |
| Occupation |  |  |  |  |  |  |  |  |  |  |  |  |  |  |
| Unemployed | <0.001 | 0.753* | 0.697 | 0.814 |  | <0.001 | 0.722* | 0.672 | 0.776 |  | 0.190 | 0.960 | 0.904 | 1.020 |
| Self-employed | 0.093 | 0.939 | 0.873 | 1.011 |  | 0.328 | 0.965 | 0.897 | 1.037 |  | <0.001 | 0.846* | 0.798 | 0.896 |
| Worker (referent) |  |  |  |  |  |  |  |  |  |  |  |  |  |  |

*Significant at p <0.05 using binary logistic regression test

Table 2. Multivariate Analysis between Openness with Eating Habits and Control Variables (cont’d)

| Variables | Eating Habits | | | |
| --- | --- | --- | --- | --- |
|  | Sweet snacks (*wajik, geplak*, donuts, wafers, chocolate, etc) | | | |
|  | p-value | OR | 95% CI | |
|  |  |  | Lower | Upper |
| Openness | <0.001 | 1.071* | 1.054 | 1.087 |
| Age | <0.001 | 0.973* | 0.970 | 0.976 |
| Gender |  |  |  |  |
| Male | <0.001 | 2.026* | 1.901 | 2.159 |
| Female (referent) |  |  |  |  |
| Marital status |  |  |  |  |
| Married/living together | <0.001 | 0.647* | 0.596 | 0.703 |
| Divorced/separated | 0.028 | 0.833* | 0.707 | 0.980 |
| Never married (referent) |  |  |  |  |
| Education |  |  |  |  |
| Not attending school | <0.001 | 0.622* | 0.489 | 0.792 |
| Attending school (referent) |  |  |  |  |
| Occupation |  |  |  |  |
| Unemployed | <0.001 | 0.753* | 0.697 | 0.814 |
| Self-employed | 0.093 | 0.939 | 0.873 | 1.011 |
| Worker (referent) |  |  |  |  |

*Significant at p <0.05 using binary logistic regression test

Table 3. Multivariate Analysis between Conscientiousness with Eating Habits and Control Variables

| Variables | Eating Habits | | | | | | | | | | | | | |
| --- | --- | --- | --- | --- | --- | --- | --- | --- | --- | --- | --- | --- | --- | --- |
|  | Sweet potatoes | | | |  | Eggs | | | |  | Fish | | | |
|  | p-value | OR | 95% CI | |  | p-value | OR | 95% CI | |  | p-value | OR | 95% CI | |
|  |  |  | Lower | Upper |  |  |  | Lower | Upper |  |  |  | Lower | Upper |
| Conscientiousness | 0.019 | 1.018* | 1.003 | 1.032 |  | <0.001 | 1.049* | 1.030 | 1.068 |  | <0.001 | 1.054* | 1.037 | 1.072 |
| Age | <0.001 | 1.018* | 1.016 | 1.020 |  | <0.001 | 0.984* | 0.981 | 0.986 |  | 0.026 | 0.997* | 0.995 | 1.000 |
| Gender |  |  |  |  |  |  |  |  |  |  |  |  |  |  |
| Male | <0.001 | 0.883* | 0.839 | 0.930 |  | 0.057 | 0.939 | 0.879 | 1.002 |  | 0.855 | 1.006 | 0.946 | 1.070 |
| Female (referent) |  |  |  |  |  |  |  |  |  |  |  |  |  |  |
| Marital status |  |  |  |  |  |  |  |  |  |  |  |  |  |  |
| Married/living together | 0.006 | 1.111* | 1.031 | 1.198 |  | <0.001 | 1.206* | 1.096 | 1.326 |  | <0.001 | 1.325* | 1.217 | 1.442 |
| Divorced/separated | 0.132 | 0.909 | 0.803 | 1.029 |  | 0.891 | 0.990 | 0.853 | 1.149 |  | 0.577 | 1.041 | 0.903 | 1.201 |
| Never married (referent) |  |  |  |  |  |  |  |  |  |  |  |  |  |  |
| Education |  |  |  |  |  |  |  |  |  |  |  |  |  |  |
| Not attending school | <0.001 | 0.649* | 0.570 | 0.738 |  | <0.001 | 0.448* | 0.393 | 0.510 |  | <0.001 | 0.719* | 0.624 | 0.828 |
| Attending school (referent) |  |  |  |  |  |  |  |  |  |  |  |  |  |  |
| Occupation |  |  |  |  |  |  |  |  |  |  |  |  |  |  |
| Unemployed | <0.001 | 0.881* | 0.828 | 0.937 |  | 0.037 | 0.921* | 0.853 | 0.995 |  | <0.001 | 0.795* | 0.741 | 0.853 |
| Self-employed | 0.136 | 1.045 | 0.986 | 1.107 |  | 0.024 | 0.919* | 0.854 | 0.989 |  | 0.059 | 1.072 | 0.997 | 1.153 |
| Worker (referent) |  |  |  |  |  |  |  |  |  |  |  |  |  |  |

*Significant at p <0.05 using binary logistic regression test

Table 3. Multivariate Analysis between Conscientiousness with Eating Habits and Control Variables (cont’d)

| Variables | Eating Habits | | | | | | | | | | | | | |
| --- | --- | --- | --- | --- | --- | --- | --- | --- | --- | --- | --- | --- | --- | --- |
|  | Meat (beef, chicken, pork, etc) | | | |  | Dairy | | | |  | Green leafy vegetables | | | |
|  | p-value | OR | 95% CI | |  | p-value | OR | 95% CI | |  | p-value | OR | 95% CI | |
|  |  |  | Lower | Upper |  |  |  | Lower | Upper |  |  |  | Lower | Upper |
| Conscientiousness | <0.001 | 1.044* | 1.030 | 1.059 |  | <0.001 | 1.030* | 1.015 | 1.046 |  | <0.001 | 1.101* | 1.079 | 1.124 |
| Age | 0.029 | 0.998* | 0.996 | 1.000 |  | 0.384 | 0.999 | 0.997 | 1.001 |  | 0.015 | 0.996* | 0.993 | 0.999 |
| Gender |  |  |  |  |  |  |  |  |  |  |  |  |  |  |
| Male | 0.007 | 1.073* | 1.020 | 1.129 |  | <0.001 | 1.163* | 1.104 | 1.226 |  | <0.001 | 0.806* | 0.748 | 0.869 |
| Female (referent) |  |  |  |  |  |  |  |  |  |  |  |  |  |  |
| Marital status |  |  |  |  |  |  |  |  |  |  |  |  |  |  |
| Married/living together | <0.001 | 0.748* | 0.695 | 0.805 |  | <0.001 | 0.611* | 0.568 | 0.657 |  | <0.001 | 1.570* | 1.417 | 1.740 |
| Divorced/separated | <0.001 | 0.607* | 0.538 | 0.686 |  | <0.001 | 0.681* | 0.600 | 0.774 |  | 0.162 | 1.130 | 0.952 | 1.342 |
| Never married (referent) |  |  |  |  |  |  |  |  |  |  |  |  |  |  |
| Education |  |  |  |  |  |  |  |  |  |  |  |  |  |  |
| Not attending school | <0.001 | 0.361* | 0.317 | 0.410 |  | <0.001 | 0.366* | 0.307 | 0.436 |  | <0.001 | 0.644* | 0.546 | 0.759 |
| Attending school (referent) |  |  |  |  |  |  |  |  |  |  |  |  |  |  |
| Occupation |  |  |  |  |  |  |  |  |  |  |  |  |  |  |
| Unemployed | <0.001 | 0.884* | 0.832 | 0.938 |  | 0.152 | 0.956 | 0.898 | 1.017 |  | <0.001 | 0.842* | 0.772 | 0.919 |
| Self-employed | 0.045 | 0.943* | 0.890 | 0.999 |  | 0.070 | 0.946 | 0.890 | 1.005 |  | 0.508 | 0.971 | 0.889 | 1.060 |
| Worker (referent) |  |  |  |  |  |  |  |  |  |  |  |  |  |  |

*Significant at p <0.05 using binary logistic regression test

Table 3. Multivariate Analysis between Conscientiousness with Eating Habits and Control Variables (cont’d)

| Variables | Eating Habits | | | | | | | | | | | | | |
| --- | --- | --- | --- | --- | --- | --- | --- | --- | --- | --- | --- | --- | --- | --- |
|  | Banana | | | |  | Papaya | | | |  | Carrot | | | |
|  | p-value | OR | 95% CI | |  | p-value | OR | 95% CI | |  | p-value | OR | 95% CI | |
|  |  |  | Lower | Upper |  |  |  | Lower | Upper |  |  |  | Lower | Upper |
| Conscientiousness | <0.001 | 1.027* | 1.012 | 1.041 |  | <0.001 | 1.030* | 1.014 | 1.047 |  | <0.001 | 1.051* | 1.036 | 1.067 |
| Age | <0.001 | 1.024* | 1.022 | 1.026 |  | <0.001 | 1.016* | 1.013 | 1.018 |  | <0.001 | 1.012* | 1.010 | 1.014 |
| Gender |  |  |  |  |  |  |  |  |  |  |  |  |  |  |
| Male | 0.772 | 0.993 | 0.945 | 1.043 |  | <0.001 | 0.804* | 0.760 | 0.850 |  | <0.001 | 0.706* | 0.670 | 0.743 |
| Female (referent) |  |  |  |  |  |  |  |  |  |  |  |  |  |  |
| Marital status |  |  |  |  |  |  |  |  |  |  |  |  |  |  |
| Married/living together | 0.066 | 0.937 | 0.873 | 1.004 |  | 0.025 | 1.099* | 1.012 | 1.194 |  | 0.059 | 1.074 | 0.997 | 1.156 |
| Divorced/separated | 0.005 | 0.841* | 0.746 | 0.948 |  | 0.144 | 0.904 | 0.790 | 1.035 |  | 0.608 | 0.968 | 0.855 | 1.096 |
| Never married (referent) |  |  |  |  |  |  |  |  |  |  |  |  |  |  |
| Education |  |  |  |  |  |  |  |  |  |  |  |  |  |  |
| Not attending school | <0.001 | 0.566* | 0.500 | 0.640 |  | <0.001 | 0.639* | 0.554 | 0.738 |  | <0.001 | 0.374* | 0.323 | 0.433 |
| Attending school (referent) |  |  |  |  |  |  |  |  |  |  |  |  |  |  |
| Occupation |  |  |  |  |  |  |  |  |  |  |  |  |  |  |
| Unemployed | 0.001 | 0.908* | 0.856 | 0.962 |  | 0.270 | 0.963 | 0.902 | 1.029 |  | 0.003 | 1.096* | 1.032 | 1.164 |
| Self-employed | 0.001 | 1.099* | 1.039 | 1.163 |  | 0.992 | 1.000 | 0.939 | 1.065 |  | 0.003 | 0.915* | 0.863 | 0.970 |
| Worker (referent) |  |  |  |  |  |  |  |  |  |  |  |  |  |  |

*Significant at p <0.05 using binary logistic regression test

Table 3. Multivariate Analysis between Conscientiousness with Eating Habits and Control Variables (cont’d)

| Variables | Eating Habits | | | | | | | | | | | | | |
| --- | --- | --- | --- | --- | --- | --- | --- | --- | --- | --- | --- | --- | --- | --- |
|  | Fast food | | | |  | Soft drink (Coca cola, sprite, etc) | | | |  | *Sambal* | | | |
|  | p-value | OR | 95% CI | |  | p-value | OR | 95% CI | |  | p-value | OR | 95% CI | |
|  |  |  | Lower | Upper |  |  |  | Lower | Upper |  |  |  | Lower | Upper |
| Conscientiousness | 0.238 | 1.013 | 0.991 | 1.036 |  | 0.872 | 0.999 | 0.981 | 1.016 |  | <0.001 | 1.042* | 1.024 | 1.060 |
| Age | <0.001 | 0.976* | 0.972 | 0.980 |  | <0.001 | 0.972* | 0.969 | 0.975 |  | <0.001 | 0.986* | 0.984 | 0.988 |
| Gender |  |  |  |  |  |  |  |  |  |  |  |  |  |  |
| Male | <0.001 | 0.790* | 0.731 | 0.854 |  | <0.001 | 2.059* | 1.933 | 2.194 |  | 0.764 | 0.990 | 0.930 | 1.055 |
| Female (referent) |  |  |  |  |  |  |  |  |  |  |  |  |  |  |
| Marital status |  |  |  |  |  |  |  |  |  |  |  |  |  |  |
| Married/living together | <0.001 | 0.682* | 0.617 | 0.755 |  | <0.001 | 0.641* | 0.590 | 0.697 |  | 0.002 | 1.154* | 1.054 | 1.263 |
| Divorced/separated | 0.019 | 0.789* | 0.647 | 0.963 |  | 0.013 | 0.813* | 0.691 | 0.957 |  | 0.419 | 0.942 | 0.816 | 1.088 |
| Never married (referent) |  |  |  |  |  |  |  |  |  |  |  |  |  |  |
| Education |  |  |  |  |  |  |  |  |  |  |  |  |  |  |
| Not attending school | <0.001 | 0.270* | 0.179 | 0.407 |  | <0.001 | 0.592* | 0.465 | 0.753 |  | <0.001 | 0.701* | 0.612 | 0.803 |
| Attending school (referent) |  |  |  |  |  |  |  |  |  |  |  |  |  |  |
| Occupation |  |  |  |  |  |  |  |  |  |  |  |  |  |  |
| Unemployed | 0.186 | 0.942 | 0.862 | 1.029 |  | <0.001 | 0.747* | 0.691 | 0.807 |  | <0.001 | 0.726* | 0.675 | 0.780 |
| Self-employed | 0.287 | 0.949 | 0.863 | 1.045 |  | 0.134 | 0.946 | 0.879 | 1.017 |  | 0.423 | 0.971 | 0.903 | 1.044 |
| Worker (referent) |  |  |  |  |  |  |  |  |  |  |  |  |  |  |

*Significant at p <0.05 using binary logistic regression test

Table 3. Multivariate Analysis between Conscientiousness with Eating Habits and Control Variables (cont’d)

| Variables | Eating Habits | | | |
| --- | --- | --- | --- | --- |
|  | Sweet snacks (*wajik, geplak*, donuts, wafers, chocolate, etc) | | | |
|  | p-value | OR | 95% CI | |
|  |  |  | Lower | Upper |
| Conscientiousness | 0.194 | 1.009 | 0.995 | 1.024 |
| Age | <0.001 | 0.986* | 0.984 | 0.988 |
| Gender |  |  |  |  |
| Male | <0.001 | 0.727* | 0.692 | 0.765 |
| Female (referent) |  |  |  |  |
| Marital status |  |  |  |  |
| Married/living together | <0.001 | 0.563* | 0.524 | 0.605 |
| Divorced/separated | <0.001 | 0.554* | 0.491 | 0.625 |
| Never married (referent) |  |  |  |  |
| Education |  |  |  |  |
| Not attending school | <0.001 | 0.426* | 0.372 | 0.488 |
| Attending school (referent) |  |  |  |  |
| Occupation |  |  |  |  |
| Unemployed | 0.012 | 1.079* | 1.017 | 1.145 |
| Self-employed | <0.001 | 1.129* | 1.068 | 1.195 |
| Worker (referent) |  |  |  |  |

*Significant at p <0.05 using binary logistic regression test

Table 4. Multivariate Analysis between Extraversion with Eating Habits and Control Variables

| Variables | Eating Habits | | | | | | | | | | | | | |
| --- | --- | --- | --- | --- | --- | --- | --- | --- | --- | --- | --- | --- | --- | --- |
|  | Sweet potatoes | | | |  | Eggs | | | |  | Fish | | | |
|  | p-value | OR | 95% CI | |  | p-value | OR | 95% CI | |  | p-value | OR | 95% CI | |
|  |  |  | Lower | Upper |  |  |  | Lower | Upper |  |  |  | Lower | Upper |
| Extraversion | <0.001 | 1.023* | 1.011 | 1.036 |  | <0.001 | 1.061* | 1.046 | 1.078 |  | 0.110 | 1.012 | 0.997 | 1.026 |
| Age | <0.001 | 1.018* | 1.016 | 1.021 |  | <0.001 | 0.984* | 0.982 | 0.987 |  | 0.054 | 0.998 | 0.995 | 1.000 |
| Gender |  |  |  |  |  |  |  |  |  |  |  |  |  |  |
| Male | <0.001 | 0.894* | 0.849 | 0.941 |  | 0.320 | 0.967 | 0.906 | 1.033 |  | 0.663 | 1.014 | 0.953 | 1.079 |
| Female (referent) |  |  |  |  |  |  |  |  |  |  |  |  |  |  |
| Marital status |  |  |  |  |  |  |  |  |  |  |  |  |  |  |
| Married/living together | 0.003 | 1.120* | 1.039 | 1.207 |  | <0.001 | 1.232* | 1.120 | 1.354 |  | <0.001 | 1.356* | 1.246 | 1.476 |
| Divorced/separated | 0.172 | 0.917 | 0.810 | 1.038 |  | 0.859 | 1.014 | 0.873 | 1.176 |  | 0.433 | 1.059 | 0.918 | 1.220 |
| Never married (referent) |  |  |  |  |  |  |  |  |  |  |  |  |  |  |
| Education |  |  |  |  |  |  |  |  |  |  |  |  |  |  |
| Not attending school | <0.001 | 0.655* | 0.576 | 0.746 |  | <0.001 | 0.459* | 0.403 | 0.523 |  | <0.001 | 0.713* | 0.619 | 0.821 |
| Attending school (referent) |  |  |  |  |  |  |  |  |  |  |  |  |  |  |
| Occupation |  |  |  |  |  |  |  |  |  |  |  |  |  |  |
| Unemployed | <0.001 | 0.879* | 0.827 | 0.935 |  | 0.022 | 0.914* | 0.846 | 0.987 |  | <0.001 | 0.782* | 0.729 | 0.839 |
| Self-employed | 0.179 | 1.040 | 0.982 | 1.102 |  | 0.010 | 0.908* | 0.844 | 0.977 |  | 0.080 | 1.067 | 0.992 | 1.147 |
| Worker (referent) |  |  |  |  |  |  |  |  |  |  |  |  |  |  |

*Significant at p <0.05 using binary logistic regression test

Table 4. Multivariate Analysis between Extraversion with Eating Habits and Control Variables (cont’d)

| Variables | Eating Habits | | | | | | | | | | | | | |
| --- | --- | --- | --- | --- | --- | --- | --- | --- | --- | --- | --- | --- | --- | --- |
|  | Meat (beef, chicken, pork, etc) | | | |  | Dairy | | | |  | Green leafy vegetables | | | |
|  | p-value | OR | 95% CI | |  | p-value | OR | 95% CI | |  | p-value | OR | 95% CI | |
|  |  |  | Lower | Upper |  |  |  | Lower | Upper |  |  |  | Lower | Upper |
| Extraversion | <0.001 | 1.070* | 1.057 | 1.082 |  | <0.001 | 1.052* | 1.039 | 1.065 |  | <0.001 | 1.034* | 1.016 | 1.052 |
| Age | 0.104 | 0.998 | 0.996 | 1.000 |  | 0.651 | 1.000 | 0.997 | 1.002 |  | 0.051 | 0.997 | 0.994 | 1.000 |
| Gender |  |  |  |  |  |  |  |  |  |  |  |  |  |  |
| Male | <0.001 | 1.110* | 1.055 | 1.169 |  | <0.001 | 1.193* | 1.132 | 1.258 |  | <0.001 | 0.823* | 0.762 | 0.887 |
| Female (referent) |  |  |  |  |  |  |  |  |  |  |  |  |  |  |
| Marital status |  |  |  |  |  |  |  |  |  |  |  |  |  |  |
| Married/living together | <0.001 | 0.763* | 0.709 | 0.821 |  | <0.001 | 0.619* | 0.575 | 0.665 |  | <0.001 | 1.637* | 1.478 | 1.813 |
| Divorced/separated | <0.001 | 0.622* | 0.551 | 0.702 |  | <0.001 | 0.692* | 0.609 | 0.787 |  | 0.078 | 1.167 | 0.983 | 1.385 |
| Never married (referent) |  |  |  |  |  |  |  |  |  |  |  |  |  |  |
| Education |  |  |  |  |  |  |  |  |  |  |  |  |  |  |
| Not attending school | <0.001 | 0.371* | 0.327 | 0.422 |  | <0.001 | 0.374* | 0.314 | 0.446 |  | <0.001 | 0.639* | 0.542 | 0.753 |
| Attending school (referent) |  |  |  |  |  |  |  |  |  |  |  |  |  |  |
| Occupation |  |  |  |  |  |  |  |  |  |  |  |  |  |  |
| Unemployed | <0.001 | 0.880* | 0.829 | 0.934 |  | 0.124 | 0.953 | 0.895 | 1.013 |  | <0.001 | 0.817* | 0.749 | 0.891 |
| Self-employed | 0.015 | 0.931* | 0.879 | 0.987 |  | 0.036 | 0.937* | 0.882 | 0.996 |  | 0.363 | 0.960 | 0.879 | 1.048 |
| Worker (referent) |  |  |  |  |  |  |  |  |  |  |  |  |  |  |

*Significant at p <0.05 using binary logistic regression test

Table 4. Multivariate Analysis between Extraversion with Eating Habits and Control Variables (cont’d)

| Variables | Eating Habits | | | | | | | | | | | | | |
| --- | --- | --- | --- | --- | --- | --- | --- | --- | --- | --- | --- | --- | --- | --- |
|  | Banana | | | |  | Papaya | | | |  | Carrot | | | |
|  | p-value | OR | 95% CI | |  | p-value | OR | 95% CI | |  | p-value | OR | 95% CI | |
|  |  |  | Lower | Upper |  |  |  | Lower | Upper |  |  |  | Lower | Upper |
| Extraversion | <0.001 | 1.035* | 1.023 | 1.047 |  | <0.001 | 1.030* | 1.017 | 1.044 |  | <0.001 | 1.030* | 1.018 | 1.043 |
| Age | <0.001 | 1.024* | 1.022 | 1.026 |  | <0.001 | 1.016* | 1.014 | 1.018 |  | <0.001 | 1.012* | 1.010 | 1.014 |
| Gender |  |  |  |  |  |  |  |  |  |  |  |  |  |  |
| Male | 0.680 | 1.010 | 0.962 | 1.062 |  | <0.001 | 0.817* | 0.772 | 0.864 |  | <0.001 | 0.718* | 0.682 | 0.756 |
| Female (referent) |  |  |  |  |  |  |  |  |  |  |  |  |  |  |
| Marital status |  |  |  |  |  |  |  |  |  |  |  |  |  |  |
| Married/living together | 0.130 | 0.948 | 0.884 | 1.016 |  | 0.010 | 1.114* | 1.026 | 1.210 |  | 0.013 | 1.098* | 1.020 | 1.182 |
| Divorced/separated | 0.009 | 0.852* | 0.756 | 0.961 |  | 0.208 | 0.917 | 0.801 | 1.050 |  | 0.835 | 0.987 | 0.872 | 1.117 |
| Never married (referent) |  |  |  |  |  |  |  |  |  |  |  |  |  |  |
| Education |  |  |  |  |  |  |  |  |  |  |  |  |  |  |
| Not attending school | <0.001 | 0.574* | 0.507 | 0.650 |  | <0.001 | 0.646* | 0.560 | 0.745 |  | <0.001 | 0.376* | 0.325 | 0.435 |
| Attending school (referent) |  |  |  |  |  |  |  |  |  |  |  |  |  |  |
| Occupation |  |  |  |  |  |  |  |  |  |  |  |  |  |  |
| Unemployed | 0.001 | 0.904* | 0.853 | 0.958 |  | 0.211 | 0.959 | 0.897 | 1.024 |  | 0.009 | 1.083* | 1.021 | 1.150 |
| Self-employed | 0.002 | 1.092* | 1.033 | 1.156 |  | 0.861 | 0.994 | 0.934 | 1.059 |  | 0.001 | 0.908* | 0.857 | 0.964 |
| Worker (referent) |  |  |  |  |  |  |  |  |  |  |  |  |  |  |

*Significant at p <0.05 using binary logistic regression test

Table 4. Multivariate Analysis between Extraversion with Eating Habits and Control Variables (cont’d)

| Variables | Eating Habits | | | | | | | | | | | | | |
| --- | --- | --- | --- | --- | --- | --- | --- | --- | --- | --- | --- | --- | --- | --- |
|  | Mango | | | |  | Instant noodle | | | |  | Fast food | | | |
|  | p-value | OR | 95% CI | |  | p-value | OR | 95% CI | |  | p-value | OR | 95% CI | |
|  |  |  | Lower | Upper |  |  |  | Lower | Upper |  |  |  | Lower | Upper |
| Extraversion | <0.001 | 1.023* | 1.011 | 1.036 |  | <0.001 | 1.023* | 1.011 | 1.036 |  | <0.001 | 1.081* | 1.062 | 1.101 |
| Age | 0.168 | 0.999 | 0.996 | 1.001 |  | <0.001 | 0.967* | 0.965 | 0.969 |  | <0.001 | 0.976* | 0.973 | 0.980 |
| Gender |  |  |  |  |  |  |  |  |  |  |  |  |  |  |
| Male | 0.002 | 0.920* | 0.874 | 0.969 |  | <0.001 | 1.108* | 1.051 | 1.168 |  | <0.001 | 0.821* | 0.759 | 0.888 |
| Female (referent) |  |  |  |  |  |  |  |  |  |  |  |  |  |  |
| Marital status |  |  |  |  |  |  |  |  |  |  |  |  |  |  |
| Married/living together | 0.741 | 0.988 | 0.919 | 1.062 |  | <0.001 | 1.282* | 1.187 | 1.384 |  | <0.001 | 0.688* | 0.622 | 0.761 |
| Divorced/separated | 0.225 | 1.079 | 0.954 | 1.220 |  | <0.001 | 1.292* | 1.139 | 1.464 |  | 0.032 | 0.804* | 0.659 | 0.981 |
| Never married (referent) |  |  |  |  |  |  |  |  |  |  |  |  |  |  |
| Education |  |  |  |  |  |  |  |  |  |  |  |  |  |  |
| Not attending school | 0.132 | 0.904 | 0.794 | 1.031 |  | <0.001 | 0.762* | 0.672 | 0.864 |  | <0.001 | 0.283* | 0.188 | 0.428 |
| Attending school (referent) |  |  |  |  |  |  |  |  |  |  |  |  |  |  |
| Occupation |  |  |  |  |  |  |  |  |  |  |  |  |  |  |
| Unemployed | 0.782 | 0.992 | 0.933 | 1.053 |  | 0.240 | 0.963 | 0.905 | 1.025 |  | 0.224 | 0.947 | 0.867 | 1.034 |
| Self-employed | 0.003 | 1.092* | 1.030 | 1.158 |  | 0.754 | 0.991 | 0.934 | 1.051 |  | 0.191 | 0.938 | 0.853 | 1.032 |
| Worker (referent) |  |  |  |  |  |  |  |  |  |  |  |  |  |  |

*Significant at p <0.05 using binary logistic regression test

Table 4. Multivariate Analysis between Extraversion with Eating Habits and Control Variables (cont’d)

| Variables | Eating Habits | | | | | | | | | | | | | |
| --- | --- | --- | --- | --- | --- | --- | --- | --- | --- | --- | --- | --- | --- | --- |
|  | Soft drink (Coca cola, sprite, etc) | | | |  | *Sambal* | | | |  | Fried snacks (*tempe, tahu, bakwan*, etc) | | | |
|  | p-value | OR | 95% CI | |  | p-value | OR | 95% CI | |  | p-value | OR | 95% CI | |
|  |  |  | Lower | Upper |  |  |  | Lower | Upper |  |  |  | Lower | Upper |
| Extraversion | <0.001 | 1.054* | 1.039 | 1.070 |  | <0.001 | 1.064* | 1.048 | 1.079 |  | <0.001 | 1.026* | 1.013 | 1.038 |
| Age | <0.001 | 0.972* | 0.969 | 0.975 |  | <0.001 | 0.986* | 0.984 | 0.989 |  | <0.001 | 0.995* | 0.993 | 0.997 |
| Gender |  |  |  |  |  |  |  |  |  |  |  |  |  |  |
| Male | <0.001 | 2.114* | 1.983 | 2.254 |  | 0.517 | 1.021 | 0.958 | 1.088 |  | <0.001 | 1.099* | 1.044 | 1.158 |
| Female (referent) |  |  |  |  |  |  |  |  |  |  |  |  |  |  |
| Marital status |  |  |  |  |  |  |  |  |  |  |  |  |  |  |
| Married/living together | <0.001 | 0.641* | 0.590 | 0.696 |  | <0.001 | 1.177* | 1.076 | 1.288 |  | 0.005 | 0.901* | 0.837 | 0.969 |
| Divorced/separated | 0.017 | 0.820* | 0.696 | 0.965 |  | 0.624 | 0.965 | 0.835 | 1.114 |  | 0.323 | 0.940 | 0.831 | 1.063 |
| Never married (referent) |  |  |  |  |  |  |  |  |  |  |  |  |  |  |
| Education |  |  |  |  |  |  |  |  |  |  |  |  |  |  |
| Not attending school | <0.001 | 0.614* | 0.483 | 0.782 |  | <0.001 | 0.721* | 0.629 | 0.825 |  | <0.001 | 0.587* | 0.519 | 0.664 |
| Attending school (referent) |  |  |  |  |  |  |  |  |  |  |  |  |  |  |
| Occupation |  |  |  |  |  |  |  |  |  |  |  |  |  |  |
| Unemployed | <0.001 | 0.751* | 0.695 | 0.811 |  | <0.001 | 0.722* | 0.672 | 0.776 |  | 0.199 | 0.961 | 0.905 | 1.021 |
| Self-employed | 0.093 | 0.939 | 0.873 | 1.010 |  | 0.262 | 0.960 | 0.893 | 1.031 |  | <0.001 | 0.844* | 0.797 | 0.894 |
| Worker (referent) |  |  |  |  |  |  |  |  |  |  |  |  |  |  |

*Significant at p <0.05 using binary logistic regression test

Table 4. Multivariate Analysis between Extraversion with Eating Habits and Control Variables (cont’d)

| Variables | Eating Habits | | | |
| --- | --- | --- | --- | --- |
|  | Sweet snacks (*wajik, geplak*, donuts, wafers, chocolate, etc) | | | |
|  | p-value | OR | 95% CI | |
|  |  |  | Lower | Upper |
| Extraversion | <0.001 | 1.032* | 1.020 | 1.044 |
| Age | <0.001 | 0.986* | 0.984 | 0.988 |
| Gender |  |  |  |  |
| Male | <0.001 | 0.739* | 0.702 | 0.777 |
| Female (referent) |  |  |  |  |
| Marital status |  |  |  |  |
| Married/living together | <0.001 | 0.565* | 0.526 | 0.608 |
| Divorced/separated | <0.001 | 0.558* | 0.494 | 0.630 |
| Never married (referent) |  |  |  |  |
| Education |  |  |  |  |
| Not attending school | <0.001 | 0.434* | 0.379 | 0.498 |
| Attending school (referent) |  |  |  |  |
| Occupation |  |  |  |  |
| Unemployed | 0.010 | 1.081* | 1.019 | 1.147 |
| Self-employed | <0.001 | 1.124* | 1.062 | 1.189 |
| Worker (referent) |  |  |  |  |

*Significant at p <0.05 using binary logistic regression test

Table 5. Multivariate Analysis between Agreeableness with Eating Habits and Control Variables

| Variables | Eating Habits | | | | | | | | | | | | | |
| --- | --- | --- | --- | --- | --- | --- | --- | --- | --- | --- | --- | --- | --- | --- |
|  | Fish | | | |  | Green leafy vegetables | | | |  | Banana | | | |
|  | p-value | OR | 95% CI | |  | p-value | OR | 95% CI | |  | p-value | OR | 95% CI | |
|  |  |  | Lower | Upper |  |  |  | Lower | Upper |  |  |  | Lower | Upper |
| Agreeableness | 0.003 | 1.028* | 1.010 | 1.047 |  | <0.001 | 1.041* | 1.018 | 1.064 |  | 0.012 | 1.019* | 1.004 | 1.034 |
| Age | 0.035 | 0.997* | 0.995 | 1.000 |  | 0.027 | 0.997* | 0.994 | 1.000 |  | <0.001 | 1.024* | 1.022 | 1.026 |
| Gender |  |  |  |  |  |  |  |  |  |  |  |  |  |  |
| Male | 0.835 | 1.007 | 0.946 | 1.071 |  | <0.001 | 0.808* | 0.749 | 0.871 |  | 0.777 | 0.993 | 0.945 | 1.043 |
| Female (referent) |  |  |  |  |  |  |  |  |  |  |  |  |  |  |
| Marital status |  |  |  |  |  |  |  |  |  |  |  |  |  |  |
| Married/living together | <0.001 | 1.352* | 1.242 | 1.471 |  | <0.001 | 1.629* | 1.471 | 1.805 |  | 0.112 | 0.945 | 0.882 | 1.013 |
| Divorced/separated | 0.476 | 1.053 | 0.913 | 1.214 |  | 0.099 | 1.155 | 0.973 | 1.371 |  | 0.006 | 0.845* | 0.750 | 0.953 |
| Never married (referent) |  |  |  |  |  |  |  |  |  |  |  |  |  |  |
| Education |  |  |  |  |  |  |  |  |  |  |  |  |  |  |
| Not attending school | <0.001 | 0.710* | 0.616 | 0.818 |  | <0.001 | 0.629* | 0.533 | 0.741 |  | <0.001 | 0.562* | 0.497 | 0.636 |
| Attending school (referent) |  |  |  |  |  |  |  |  |  |  |  |  |  |  |
| Occupation |  |  |  |  |  |  |  |  |  |  |  |  |  |  |
| Unemployed | <0.001 | 0.782* | 0.729 | 0.838 |  | <0.001 | 0.815* | 0.748 | 0.889 |  | <0.001 | 0.900* | 0.850 | 0.954 |
| Self-employed | 0.077 | 1.067 | 0.993 | 1.147 |  | 0.405 | 0.963 | 0.882 | 1.052 |  | 0.001 | 1.097* | 1.037 | 1.160 |
| Worker (referent) |  |  |  |  |  |  |  |  |  |  |  |  |  |  |

*Significant at p <0.05 using binary logistic regression test

Table 5. Multivariate Analysis between Agreeableness with Eating Habits and Control Variables (cont’d)

| Variables | Eating Habits | | | | | | | | | | | | | |
| --- | --- | --- | --- | --- | --- | --- | --- | --- | --- | --- | --- | --- | --- | --- |
|  | Papaya | | | |  | Carrot | | | |  | Mango | | | |
|  | p-value | OR | 95% CI | |  | p-value | OR | 95% CI | |  | p-value | OR | 95% CI | |
|  |  |  | Lower | Upper |  |  |  | Lower | Upper |  |  |  | Lower | Upper |
| Agreeableness | 0.023 | 1.020* | 1.003 | 1.037 |  | 0.103 | 1.013 | 0.997 | 1.029 |  | 0.011 | 1.020* | 1.005 | 1.036 |
| Age | <0.001 | 1.016* | 1.013 | 1.018 |  | <0.001 | 1.012* | 1.010 | 1.014 |  | 0.111 | 0.998 | 0.996 | 1.000 |
| Gender |  |  |  |  |  |  |  |  |  |  |  |  |  |  |
| Male | <0.001 | 0.804* | 0.761 | 0.851 |  | <0.001 | 0.707* | 0.672 | 0.745 |  | <0.001 | 0.909* | 0.864 | 0.957 |
| Female (referent) |  |  |  |  |  |  |  |  |  |  |  |  |  |  |
| Marital status |  |  |  |  |  |  |  |  |  |  |  |  |  |  |
| Married/living together | 0.012 | 1.111* | 1.023 | 1.206 |  | 0.015 | 1.095* | 1.018 | 1.179 |  | 0.688 | 0.985 | 0.916 | 1.059 |
| Divorced/separated | 0.170 | 0.910 | 0.795 | 1.041 |  | 0.743 | 0.979 | 0.865 | 1.108 |  | 0.267 | 1.072 | 0.948 | 1.212 |
| Never married (referent) |  |  |  |  |  |  |  |  |  |  |  |  |  |  |
| Education |  |  |  |  |  |  |  |  |  |  |  |  |  |  |
| Not attending school | <0.001 | 0.635* | 0.550 | 0.733 |  | <0.001 | 0.369* | 0.319 | 0.427 |  | 0.088 | 0.893 | 0.783 | 1.017 |
| Attending school (referent) |  |  |  |  |  |  |  |  |  |  |  |  |  |  |
| Occupation |  |  |  |  |  |  |  |  |  |  |  |  |  |  |
| Unemployed | 0.169 | 0.955 | 0.894 | 1.020 |  | 0.013 | 1.079* | 1.016 | 1.145 |  | 0.714 | 0.989 | 0.931 | 1.050 |
| Self-employed | 0.943 | 0.998 | 0.937 | 1.062 |  | 0.002 | 0.912* | 0.860 | 0.967 |  | 0.002 | 1.095* | 1.033 | 1.160 |
| Worker (referent) |  |  |  |  |  |  |  |  |  |  |  |  |  |  |

*Significant at p <0.05 using binary logistic regression test

Table 5. Multivariate Analysis between Agreeableness with Eating Habits and Control Variables (cont’d)

| Variables | Eating Habits | | | | | | | | | | | | | |
| --- | --- | --- | --- | --- | --- | --- | --- | --- | --- | --- | --- | --- | --- | --- |
|  | Fast food | | | |  | Soft drink (Coca cola, sprite, etc) | | | |  | Fried snacks (*tempe, tahu, bakwan*, etc) | | | |
|  | p-value | OR | 95% CI | |  | p-value | OR | 95% CI | |  | p-value | OR | 95% CI | |
|  |  |  | Lower | Upper |  |  |  | Lower | Upper |  |  |  | Lower | Upper |
| Agreeableness | 0.498 | 0.992 | 0.969 | 1.015 |  | 0.024 | 0.978* | 0.960 | 0.997 |  | 0.060 | 0.985 | 0.970 | 1.001 |
| Age | <0.001 | 0.976* | 0.973 | 0.980 |  | <0.001 | 0.972* | 0.969 | 0.975 |  | <0.001 | 0.995* | 0.993 | 0.997 |
| Gender |  |  |  |  |  |  |  |  |  |  |  |  |  |  |
| Male | <0.001 | 0.791* | 0.732 | 0.855 |  | <0.001 | 2.063* | 1.936 | 2.198 |  | 0.001 | 1.088* | 1.033 | 1.145 |
| Female (referent) |  |  |  |  |  |  |  |  |  |  |  |  |  |  |
| Marital status |  |  |  |  |  |  |  |  |  |  |  |  |  |  |
| Married/living together | <0.001 | 0.686* | 0.621 | 0.758 |  | <0.001 | 0.642* | 0.591 | 0.697 |  | 0.006 | 0.902* | 0.838 | 0.971 |
| Divorced/separated | 0.021 | 0.791* | 0.648 | 0.965 |  | 0.013 | 0.814* | 0.692 | 0.958 |  | 0.297 | 0.937 | 0.828 | 1.059 |
| Never married (referent) |  |  |  |  |  |  |  |  |  |  |  |  |  |  |
| Education |  |  |  |  |  |  |  |  |  |  |  |  |  |  |
| Not attending school | <0.001 | 0.268* | 0.178 | 0.405 |  | <0.001 | 0.591* | 0.464 | 0.752 |  | <0.001 | 0.576* | 0.510 | 0.652 |
| Attending school (referent) |  |  |  |  |  |  |  |  |  |  |  |  |  |  |
| Occupation |  |  |  |  |  |  |  |  |  |  |  |  |  |  |
| Unemployed | 0.153 | 0.938 | 0.859 | 1.024 |  | <0.001 | 0.746* | 0.690 | 0.806 |  | 0.155 | 0.957 | 0.901 | 1.017 |
| Self-employed | 0.283 | 0.949 | 0.863 | 1.044 |  | 0.143 | 0.947 | 0.880 | 1.019 |  | <0.001 | 0.848* | 0.800 | 0.898 |
| Worker (referent) |  |  |  |  |  |  |  |  |  |  |  |  |  |  |

*Significant at p <0.05 using binary logistic regression test

Table 5. Multivariate Analysis between Agreeableness with Eating Habits and Control Variables (cont’d)

| Variables | Eating Habits | | | |
| --- | --- | --- | --- | --- |
|  | Sweet snacks (*wajik, geplak*, donuts, wafers, chocolate, etc) | | | |
|  | p-value | OR | 95% CI | |
|  |  |  | Lower | Upper |
| Agreeableness | 0.052 | 0.985 | 0.971 | 1.000 |
| Age | <0.001 | 0.986* | 0.984 | 0.988 |
| Gender |  |  |  |  |
| Male | <0.001 | 0.728* | 0.693 | 0.766 |
| Female (referent) |  |  |  |  |
| Marital status |  |  |  |  |
| Married/living together | <0.001 | 0.566* | 0.527 | 0.609 |
| Divorced/separated | <0.001 | 0.556* | 0.492 | 0.627 |
| Never married (referent) |  |  |  |  |
| Education |  |  |  |  |
| Not attending school | <0.001 | 0.424* | 0.370 | 0.486 |
| Attending school (referent) |  |  |  |  |
| Occupation |  |  |  |  |
| Unemployed | 0.016 | 1.075* | 1.013 | 1.141 |
| Self-employed | <0.001 | 1.130* | 1.068 | 1.195 |
| Worker (referent) |  |  |  |  |

*Significant at p <0.05 using binary logistic regression test

Table 6. Multivariate Analysis between Neuroticism with Eating Habits and Control Variables

| Variables | Eating Habits | | | | | | | | | | | | | |
| --- | --- | --- | --- | --- | --- | --- | --- | --- | --- | --- | --- | --- | --- | --- |
|  | Sweet potatoes | | | |  | Meat (beef, chicken, pork, etc) | | | |  | Dairy | | | |
|  | p-value | OR | 95% CI | |  | p-value | OR | 95% CI | |  | p-value | OR | 95% CI | |
|  |  |  | Lower | Upper |  |  |  | Lower | Upper |  |  |  | Lower | Upper |
| Neuroticism | 0.488 | 1.004 | 0.992 | 1.016 |  | <0.001 | 0.974* | 0.963 | 0.986 |  | <0.001 | 0.975* | 0.963 | 0.987 |
| Age | <0.001 | 1.018* | 1.016 | 1.020 |  | 0.021 | 0.998* | 0.996 | 1.000 |  | 0.300 | 0.999 | 0.997 | 1.001 |
| Gender |  |  |  |  |  |  |  |  |  |  |  |  |  |  |
| Male | <0.001 | 0.886* | 0.841 | 0.933 |  | 0.025 | 1.060* | 1.007 | 1.116 |  | <0.001 | 1.149* | 1.090 | 1.211 |
| Female (referent) |  |  |  |  |  |  |  |  |  |  |  |  |  |  |
| Marital status |  |  |  |  |  |  |  |  |  |  |  |  |  |  |
| Married/living together | 0.003 | 1.120* | 1.039 | 1.208 |  | <0.001 | 0.761* | 0.707 | 0.818 |  | <0.001 | 0.617* | 0.574 | 0.663 |
| Divorced/separated | 0.153 | 0.913 | 0.807 | 1.034 |  | <0.001 | 0.614* | 0.544 | 0.693 |  | <0.001 | 0.686* | 0.603 | 0.779 |
| Never married (referent) |  |  |  |  |  |  |  |  |  |  |  |  |  |  |
| Education |  |  |  |  |  |  |  |  |  |  |  |  |  |  |
| Not attending school | <0.001 | 0.645* | 0.567 | 0.734 |  | <0.001 | 0.357* | 0.314 | 0.406 |  | <0.001 | 0.363* | 0.305 | 0.433 |
| Attending school (referent) |  |  |  |  |  |  |  |  |  |  |  |  |  |  |
| Occupation |  |  |  |  |  |  |  |  |  |  |  |  |  |  |
| Unemployed | <0.001 | 0.875* | 0.823 | 0.931 |  | <0.001 | 0.875* | 0.824 | 0.929 |  | 0.100 | 0.949 | 0.892 | 1.010 |
| Self-employed | 0.145 | 1.044 | 0.985 | 1.106 |  | 0.035 | 0.940* | 0.887 | 0.996 |  | 0.058 | 0.943 | 0.888 | 1.002 |
| Worker (referent) |  |  |  |  |  |  |  |  |  |  |  |  |  |  |

*Significant at p <0.05 using binary logistic regression test

Table 6. Multivariate Analysis between Neuroticism with Eating Habits and Control Variables (cont’d)

| Variables | Eating Habits | | | | | | | | | | | | | |
| --- | --- | --- | --- | --- | --- | --- | --- | --- | --- | --- | --- | --- | --- | --- |
|  | Green leafy vegetables | | | |  | Banana | | | |  | Papaya | | | |
|  | p-value | OR | 95% CI | |  | p-value | OR | 95% CI | |  | p-value | OR | 95% CI | |
|  |  |  | Lower | Upper |  |  |  | Lower | Upper |  |  |  | Lower | Upper |
| Neuroticism | <0.001 | 0.942* | 0.925 | 0.958 |  | 0.557 | 1.003 | 0.992 | 1.015 |  | 0.072 | 0.988 | 0.975 | 1.001 |
| Age | 0.009 | 0.996* | 0.993 | 0.999 |  | <0.001 | 1.024* | 1.022 | 1.026 |  | <0.001 | 1.016* | 1.013 | 1.018 |
| Gender |  |  |  |  |  |  |  |  |  |  |  |  |  |  |
| Male | <0.001 | 0.783* | 0.726 | 0.845 |  | 0.878 | 0.996 | 0.948 | 1.047 |  | <0.001 | 0.800* | 0.756 | 0.847 |
| Female (referent) |  |  |  |  |  |  |  |  |  |  |  |  |  |  |
| Marital status |  |  |  |  |  |  |  |  |  |  |  |  |  |  |
| Married/living together | <0.001 | 1.626* | 1.468 | 1.801 |  | 0.128 | 0.947 | 0.884 | 1.016 |  | 0.012 | 1.112* | 1.024 | 1.207 |
| Divorced/separated | 0.098 | 1.156 | 0.974 | 1.372 |  | 0.006 | 0.847* | 0.752 | 0.954 |  | 0.176 | 0.911 | 0.796 | 1.043 |
| Never married (referent) |  |  |  |  |  |  |  |  |  |  |  |  |  |  |
| Education |  |  |  |  |  |  |  |  |  |  |  |  |  |  |
| Not attending school | <0.001 | 0.630* | 0.534 | 0.742 |  | <0.001 | 0.561* | 0.495 | 0.635 |  | <0.001 | 0.634* | 0.550 | 0.732 |
| Attending school (referent) |  |  |  |  |  |  |  |  |  |  |  |  |  |  |
| Occupation |  |  |  |  |  |  |  |  |  |  |  |  |  |  |
| Unemployed | <0.001 | 0.820* | 0.752 | 0.894 |  | <0.001 | 0.899* | 0.849 | 0.953 |  | 0.184 | 0.956 | 0.895 | 1.022 |
| Self-employed | 0.405 | 0.963 | 0.882 | 1.052 |  | 0.001 | 1.098* | 1.038 | 1.161 |  | 0.956 | 0.998 | 0.937 | 1.063 |
| Worker (referent) |  |  |  |  |  |  |  |  |  |  |  |  |  |  |

*Significant at p <0.05 using binary logistic regression test

Table 6. Multivariate Analysis between Neuroticism with Eating Habits and Control Variables (cont’d)

| Variables | Eating Habits | | | | | | | | | | | | | |
| --- | --- | --- | --- | --- | --- | --- | --- | --- | --- | --- | --- | --- | --- | --- |
|  | Carrot | | | |  | Mango | | | |  | Instant noodle | | | |
|  | p-value | OR | 95% CI | |  | p-value | OR | 95% CI | |  | p-value | OR | 95% CI | |
|  |  |  | Lower | Upper |  |  |  | Lower | Upper |  |  |  | Lower | Upper |
| Neuroticism | <0.001 | 0.973* | 0.962 | 0.985 |  | 0.143 | 1.009 | 0.997 | 1.021 |  | 0.012 | 1.016* | 1.004 | 1.029 |
| Age | <0.001 | 1.012* | 1.010 | 1.014 |  | 0.175 | 0.999 | 0.996 | 1.001 |  | <0.001 | 0.967* | 0.965 | 0.969 |
| Gender |  |  |  |  |  |  |  |  |  |  |  |  |  |  |
| Male | <0.001 | 0.697* | 0.662 | 0.735 |  | 0.001 | 0.915* | 0.869 | 0.963 |  | <0.001 | 1.106* | 1.049 | 1.166 |
| Female (referent) |  |  |  |  |  |  |  |  |  |  |  |  |  |  |
| Marital status |  |  |  |  |  |  |  |  |  |  |  |  |  |  |
| Married/living together | 0.017 | 1.093* | 1.016 | 1.177 |  | 0.750 | 0.988 | 0.919 | 1.063 |  | <0.001 | 1.284* | 1.189 | 1.387 |
| Divorced/separated | 0.741 | 0.979 | 0.865 | 1.108 |  | 0.251 | 1.075 | 0.950 | 1.215 |  | <0.001 | 1.288* | 1.136 | 1.460 |
| Never married (referent) |  |  |  |  |  |  |  |  |  |  |  |  |  |  |
| Education |  |  |  |  |  |  |  |  |  |  |  |  |  |  |
| Not attending school | <0.001 | 0.370* | 0.320 | 0.428 |  | 0.079 | 0.890 | 0.781 | 1.014 |  | <0.001 | 0.749* | 0.660 | 0.849 |
| Attending school (referent) |  |  |  |  |  |  |  |  |  |  |  |  |  |  |
| Occupation |  |  |  |  |  |  |  |  |  |  |  |  |  |  |
| Unemployed | 0.009 | 1.083* | 1.020 | 1.150 |  | 0.668 | 0.987 | 0.929 | 1.048 |  | 0.174 | 0.958 | 0.900 | 1.019 |
| Self-employed | 0.002 | 0.912* | 0.860 | 0.967 |  | 0.002 | 1.096* | 1.034 | 1.162 |  | 0.846 | 0.994 | 0.937 | 1.055 |
| Worker (referent) |  |  |  |  |  |  |  |  |  |  |  |  |  |  |

*Significant at p <0.05 using binary logistic regression test

Table 6. Multivariate Analysis between Neuroticism with Eating Habits and Control Variables (cont’d)

| Variables | Eating Habits | | | | | | | | | | | | | |
| --- | --- | --- | --- | --- | --- | --- | --- | --- | --- | --- | --- | --- | --- | --- |
|  | Fast food | | | |  | Soft drink (Coca cola, sprite, etc) | | | |  | Fried snacks (*tempe, tahu, bakwan,* etc) | | | |
|  | p-value | OR | 95% CI | |  | p-value | OR | 95% CI | |  | p-value | OR | 95% CI | |
|  |  |  | Lower | Upper |  |  |  | Lower | Upper |  |  |  | Lower | Upper |
| Neuroticism | 0.287 | 0.990 | 0.972 | 1.008 |  | 0.027 | 1.017* | 1.002 | 1.032 |  | 0.001 | 1.021* | 1.009 | 1.033 |
| Age | <0.001 | 0.976* | 0.972 | 0.979 |  | <0.001 | 0.972* | 0.969 | 0.975 |  | <0.001 | 0.995* | 0.993 | 0.997 |
| Gender |  |  |  |  |  |  |  |  |  |  |  |  |  |  |
| Male | <0.001 | 0.786* | 0.727 | 0.851 |  | <0.001 | 2.079* | 1.950 | 2.216 |  | <0.001 | 1.099* | 1.043 | 1.157 |
| Female (referent) |  |  |  |  |  |  |  |  |  |  |  |  |  |  |
| Marital status |  |  |  |  |  |  |  |  |  |  |  |  |  |  |
| Married/living together | <0.001 | 0.685* | 0.620 | 0.757 |  | <0.001 | 0.642* | 0.591 | 0.697 |  | 0.006 | 0.902* | 0.838 | 0.971 |
| Divorced/separated | 0.021 | 0.791* | 0.648 | 0.965 |  | 0.013 | 0.813* | 0.691 | 0.956 |  | 0.294 | 0.936 | 0.828 | 1.059 |
| Never married (referent) |  |  |  |  |  |  |  |  |  |  |  |  |  |  |
| Education |  |  |  |  |  |  |  |  |  |  |  |  |  |  |
| Not attending school | <0.001 | 0.269* | 0.178 | 0.406 |  | <0.001 | 0.591* | 0.464 | 0.752 |  | <0.001 | 0.576* | 0.509 | 0.651 |
| Attending school (referent) |  |  |  |  |  |  |  |  |  |  |  |  |  |  |
| Occupation |  |  |  |  |  |  |  |  |  |  |  |  |  |  |
| Unemployed | 0.161 | 0.939 | 0.860 | 1.025 |  | <0.001 | 0.746* | 0.691 | 0.806 |  | 0.131 | 0.954 | 0.898 | 1.014 |
| Self-employed | 0.277 | 0.948 | 0.862 | 1.043 |  | 0.136 | 0.946 | 0.879 | 1.018 |  | <0.001 | 0.847* | 0.800 | 0.898 |
| Worker (referent) |  |  |  |  |  |  |  |  |  |  |  |  |  |  |

*Significant at p <0.05 using binary logistic regression test

Table 6. Multivariate Analysis between Neuroticism with Eating Habits and Control Variables (cont’d)

| Variables | Eating Habits | | | |
| --- | --- | --- | --- | --- |
|  | Sweet snacks (*wajik, geplak*, donuts, wafers, chocolate, etc) | | | |
|  | p-value | OR | 95% CI | |
|  |  |  | Lower | Upper |
| Neuroticism | 0.018 | 1.014* | 1.002 | 1.026 |
| Age | <0.001 | 0.986* | 0.984 | 0.988 |
| Gender |  |  |  |  |
| Male | <0.001 | 0.733* | 0.697 | 0.771 |
| Female (referent) |  |  |  |  |
| Marital status |  |  |  |  |
| Married/living together | <0.001 | 0.566* | 0.527 | 0.609 |
| Divorced/separated | <0.001 | 0.555* | 0.492 | 0.626 |
| Never married (referent) |  |  |  |  |
| Education |  |  |  |  |
| Not attending school | <0.001 | 0.424* | 0.370 | 0.486 |
| Attending school (referent) |  |  |  |  |
| Occupation |  |  |  |  |
| Unemployed | 0.019 | 1.073* | 1.012 | 1.139 |
| Self-employed | <0.001 | 1.129* | 1.067 | 1.195 |
| Worker (referent) |  |  |  |  |

*Significant at p <0.05 using binary logistic regression test
